# A molecular portrait of microsatellite instability across multiple cancers

**DOI:** 10.1038/ncomms15180

**Published:** 2017-06-06

**Authors:** Isidro Cortes-Ciriano, Sejoon Lee, Woong-Yang Park, Tae-Min Kim, Peter J. Park

**Affiliations:** 1 Department of Biomedical Informatics, Harvard Medical School, Boston, Massachusetts 02115, USA; 2 Ludwig Center at Harvard, Boston, Massachusetts 02115, USA; 3 Samsung Genome Institute, Samsung Medical Center, Seoul 06351, South Korea; 4 Department of Medical Informatics and Catholic Cancer Research Institute, College of Medicine, The Catholic University of Korea, Seoul 06591, South Korea

## Abstract

Microsatellite instability (MSI) refers to the hypermutability of short repetitive sequences in the genome caused by impaired DNA mismatch repair. Although MSI has been studied for decades, large amounts of sequencing data now available allows us to examine the molecular fingerprints of MSI in greater detail. Here, we analyse ∼8,000 exomes and ∼1,000 whole genomes of cancer patients across 23 cancer types. Our analysis reveals that the frequency of MSI events is highly variable within and across tumour types. We also identify genes in DNA repair and oncogenic pathways recurrently subject to MSI and uncover non-coding loci that frequently display MSI. Finally, we propose a highly accurate exome-based predictive model for the MSI phenotype. These results advance our understanding of the genomic drivers and consequences of MSI, and our comprehensive catalogue of tumour-type-specific MSI loci will enable panel-based MSI testing to identify patients who are likely to benefit from immunotherapy.

## Abstract (web summary)

Some cancers with DNA mismatch repair deficiency display microsatellite instability. Here the authors analyse twenty three cancer types at the exome and whole-genome level, and identify loci with recurrent microsatellite instability that could be used to identify patients who would benefit from immunotherapy.

Microsatellites (MS) are tandem repeats of short DNA sequences, abundant throughout the human genome. Owing to their high mutation rates, MS have been widely used as polymorphic markers in population genetics and forensics. Microsatellite instability (MSI) is a hypermutator phenotype that occurs in tumours with impaired DNA mismatch repair (MMR) and is characterized by widespread length polymorphisms of MS repeats due to DNA polymerase slippage^1^ as well as by elevated frequency of single-nucleotide variants (SNVs). MSI in sporadic cases is caused by inactivation of MMR genes (for example, *MLH1*, *MSH2*, *MSH3*, *MSH6* and *PMS2*) through somatic mutations, with increased risk of cancer for those with inherited germline mutations (that is, Lynch syndrome)^2^. MSI also occurs by hypermethylation of the *MLH1* promoter (for example, associated with the somatic *BRAF* V600E mutation)^3^, epigenetic inactivation of *MSH2* (ref. 4), or downregulation of MMR genes by microRNAs^5^. MSI events within coding regions can alter the reading frame, leading to truncated, functionally-impaired proteins^6^.

MSI is observed in 15% of sporadic colorectal tumours diagnosed in the United States^7^, and has been reported in glioblastomas, lymphomas, stomach, urinary tract, ovarian and endometrial tumours^8^. In clinical settings, detection of MSI is customarily performed by immunohistochemical analysis of MMR proteins or by profiling the Bethesda markers^7^, which often include two mononucleotide (BAT25 and BAT26) and three dinucleotide (D5S346, D2S123 and D17S250) MS loci. Colorectal tumours unstable at >40% of the Bethesda markers are considered MSI-High (MSI-H) and are known to have a better prognosis and to be less prone to metastasis than MS stable (MSS) tumours^9^.

It was conjectured more than two decades ago that the less aggressive nature of MSI tumours may be due to their high incidence of somatic mutations, which results in a greater likelihood of having mutated genes whose products elicit antitumour immune responses^10^. Indeed, in melanoma and lung tumours, an elevated mutational load has been associated with an increased rate of response to anti-CTLA-4 and anti-PD-1 therapies, respectively, likely as a result of a higher neo-antigen burden leading to antitumour immune response^11^,^12^. Other reports have shown that colorectal patients with MMR deficiency have better responses to immunotherapy by PD-1 immune checkpoint blockade and show improved progression-free survival^13^. Although the precise link between the mutator phenotype with MSI and patient response to immunotherapy remains to be elucidated, it is clear that accurate identification of patients with the hypermutator phenotype and their genomic characterization is of therapeutic importance.

In this study, we analyse the extent and characteristics of MSI in ∼8,000 exomes and ∼1,000 whole genomes spanning 23 tumour types, utilizing data from The Cancer Genome Atlas (TCGA)^14^. This represents a major expansion of our previous MSI analysis in 277 colorectal and uterine endometrial exomes^15^ and complements a recent large-scale analysis by Hause *et al*.^16^ We systematically profile the patterns of MSI mutations in both nuclear and mitochondrial DNA, characterize the affected pathways, and find associations with epigenomic features. These analyses uncover new genes harbouring frameshift MSI events with varying degrees of cancer-type specificity and generate the most comprehensive catalogue to date of MS loci selectively subject to DNA slippage events in MSI-H tumours. This set includes loci in the non-coding portions of the genome revealed by whole-genome sequencing. Lastly, we describe highly accurate predictive models of MSI-H status based on exome data.

## Results

### The exome-wide profiles of MSI in cancer genomes

**Figure 1:**
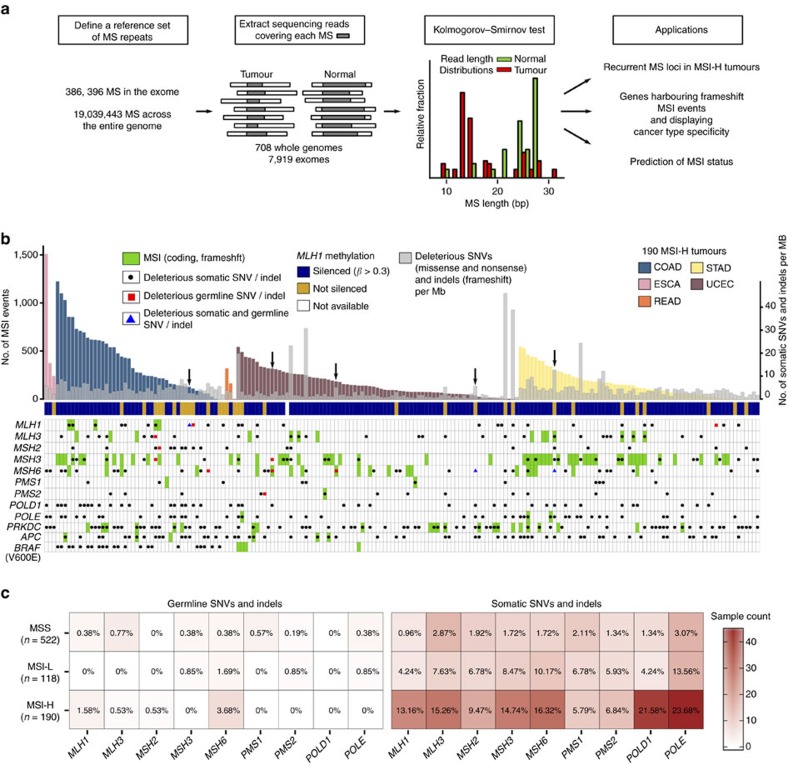
Schematic overview of the MSI calling pipeline. (**a**) A reference set of exonic and genome-wide MS repeats was assembled from the human reference genome hg19. The sequencing reads spanning each MS repeat and at least 2 base pairs at each flanking side were extracted from the tumour and normal BAM files. This process was repeated for all MS repeats in the reference sets across all pairs of matched normal-tumour samples. The Kolmogorov–Smirnov test was used to evaluate whether the read length distributions from the normal and tumour samples differed significantly (FDR<0.05). The exonic and genome-wide MSI calls served to identify MS loci recurrently altered by MSI in MSI-H tumours, discover frequent frameshift mutations and to predict MSI status. (**b**) Landscape of somatic MSI in MSI-H tumours. MSI events (frameshift and in-frame), deleterious SNV (missense, nonsense and splice site) and indel (frameshift) rates in 190 MSI-H exomes. Samples harbouring hypermethylation of the *MLH1* promoter are denoted by blue squares. Deleterious germline and somatic mutations (that is, missense, nonsense, splice site and frameshift) are depicted in black and red, respectively, whereas frameshfit MSI events are shown in green. Black arrows mark patients with germline and somatic mutations in MMR genes. (**c**) Germline and somatic mutations in MMR genes, *POLE* and *POLD1* in MSS, MSI-L and MSI-H tumours. The heatmap and the cell labels report the number and percentage of samples in each category harbouring mutations, respectively.

To obtain an MSI landscape in cancer patients, we analysed TCGA exome-sequencing data for 7,919 tumour and matched normal pairs across 23 cancer types (Table 1). We identified 386,396 microsatellite repeats among the 39,496 RefSeq mRNA sequences^15^ and tested for the presence of MSI at MS that had sufficient coverage in the exome data (Fig. 1a; Methods).

We first investigated the five tumour types for which the MSI status was determined by TCGA using capillary sequencing-based fragment length assay (COAD: colon adenocarcinoma, ESCA: oesophageal carcinoma, READ: rectal adenocarcinoma, STAD: stomach adenocarcinoma and UCEC: uterine corpus endometrial carcinoma; Supplementary Data 1)^17^,^18^,^19^. These five tumour types have been recognized as MSI-prone and contained the majority of MSI events we discovered (44,462 MSI events in these five tumour types, *n*=904, versus 29,659 events in the remaining cases, *n*=7,015). Figure 1b shows the abundance of MSI events across the 190 MSI-H cases in these five tumour types (see Supplementary Figs 1 and 2a for the remaining 118 MSI-L (MSI-Low) and 522 MSS tumour genomes in these five tumour types, and Supplementary Fig. 2b and Supplementary Data 1 for all tumour types). Our analysis confirms that MSI mutations represent a continuous rather than a dichotomous phenotype. The figure also shows a pronounced variability in the number of MSI events across MSI-H cases and across cancer types, indicating substantial intra- and inter-tumour-type heterogeneity in the genomic impact of MSI (Supplementary Fig. 1; Supplementary Data 1). For example, we note that 17% of the MSI-H tumours have fewer than 50 detected MSI events (7% with fewer than 10), including one COAD MSI-H tumour without any exonic MSI events, while others have several hundred exonic MSI events (‘exonic' regions here also include some neighbouring non-exonic elements such as untranslated regions (UTRs) and introns).

Next, we identified genes with recurrent MSI events using MutSigCV^20^. The genes displaying significant enrichment for coding MSI (false discovery rate (FDR)<0.05) along with their level of significance across three tumour types are shown in Supplementary Fig. 3. Pathway analysis reveals that transmembrane/TGFβ, response to cellular stress/DNA damage and chromosome/M-phase-related molecular functions are significantly enriched in genes harbouring recurrent MSI in COAD, STAD and UCEC cases, respectively (Supplementary Data 2; *P*<0.01).

### The mutational landscape of DNA repair pathways

The rates of deleterious mutations (for example, missense, nonsense and splicing site SNVs, and frameshift indels) and frameshift MSI events for *MLH1*, *MLH3*, *MSH2*, *MSH3*, *MSH6*, *PMS1*, *PMS2*, *POLE*, *POLD1*, *PRKDC*, *APC* and *BRAF* (p.V600E) are shown in Fig. 1b. Among these genes (selected on the basis of their association with MSI, DNA repair and colorectal cancer), MSI frameshift events represent a major source for the inactivation of *MSH3* and *MSH6*. In contrast, deleterious SNV mutations more frequently contribute to the loss of function of *POLD1* and *POLE* (27 and 23% of cases, respectively). We next examined the patterns of frameshift MSI events across MSI-prone tumours. We selected a set of 151 genes^21^ involved in several DNA repair pathways, including non-homologous end joining (NHEJ), homologous recombination (HR), base excision (BER), RecQ helicase-Like (RECQ), translesion synthesis (TLS) and ataxia telangiectasia mutated (ATM)^21^. We find that COAD samples harbouring a large number of MSI events (>500 in our samples) are enriched for *MLH1* promoter hypermethylation (Fig. 1b), as previously reported for this tumour type^15^. The genes most frequently targeted by MSI are *RAD50* (16% of MSI-H tumours), *ATR* (15%) and *RBBP8* (10%) (Supplementary Data 3; Supplementary Fig. 4a).

We have also examined the impact of germline mutations in the MSI-H cases. We observe that 4 COAD (9%), 4 UCEC (5%) and 2 STAD (3%) patients harbour deleterious germline mutations in MMR genes. Of these, at least five patients may have acquired the MSI-H phenotypes due to biallelic inactivation of MMR genes, where the inherited germline mutations of MMR genes are complemented with somatically acquired mutations of the corresponding genes. One COAD sample harboured germline and somatic mutations in *MLH1*; 1 STAD and 3 UCEC cases harbour germline and somatic mutations in *MSH6.* Overall, germline mutations in MMR genes, *POLE* and *POLD1* are consistently more prevalent in MSI-H patients compared to MSS cases (Fig. 1c; Supplementary Fig. 5). These frequencies of germline mutation carriers in MMR genes are likely to be under-estimates, since we have applied stringent filtering criteria for our germline calls (see Methods) to account for the uncertain pathogenicity of missense mutations^22^, as well as the technical challenges in identifying mutations in *PMS2*, which has multiple copies of its pseudogenes in the genome^23^.

Although it is difficult to pinpoint the genomic events initiating MMR deficiency, it is likely that truncating mutations in various MMR genes in addition to the hypermethylation of *MLH1* shape the MSI-H genomes, leading to further accumulation of mutations in the DNA repair pathway. To investigate the downstream impact of somatic alterations in MMR genes and proofreading DNA polymerases, we examined the correlation between gene expression and promoter methylation, DNA copy numbers, somatic SNVs and indels, and MSI events (Supplementary Fig. 6). For *MLH1*, only the DNA methylation level is associated with gene expression levels (r=−0.79; Pearson correlation), consistent with a previous report^3^. No apparent relationship between promoter methylation and gene expression is observed for the other genes examined. Other than *MLH1*, the most common genomic events that show association with gene expression (*P*<0.05; Mann–Whitney test) are the truncating SNVs and frameshift MSI events (*MLH3*, *MSH2*, *MSH3*, *MSH6*, *PMS1* and *POLD1*), suggesting that these somatic events are responsible for the under-expression of these genes. This may be explained by nonsense mediated decay where RNA transcripts harbouring premature terminating codons (for example, truncating SNVs and frameshift MSI) are degraded by RNA surveillance mechanisms^24^. Further investigation will be required to ascertain whether the under-expression of MMR genes associated with monoallelic truncating mutations may lead to their functional inactivation, since whether MMR mutations have haploinsufficiency (that is, heterozygous MMR mutations have functional roles) is debatable^25^. The association between DNA copy number and gene expression (*r*>0.2; Pearson correlation) is observed for *MSH2* and *POLD1*. We do not observe any significant association between gene expression and germline truncating mutations.

### Cancer-type specificity in loci targeted by frameshift MSI

We investigated the frequency of frameshift MSI events in 130 cancer-related genes^26^ across the MSI-prone tumours. Tumour-type specificity of frameshift MSI is evident for some well-known targets of MSI, such as *ACVR2A* (52% of MSI-H tumours) and *TGFBR2* (44%) (enriched in both COAD and STAD; *P*<0.05, one-tailed Fisher's exact test) as well as *RPL22* (31%), *RNF43* (31%), *MLL3* (27%), *PRDM2* (21%), *JAK1* (16%) and *APC* (3%) (Supplementary Fig. 4b; Supplementary Data 4)^27^,^28^,^29^. For instance, frameshift MSI events are present in *TGFBR2* for 26/45 (58%) of COAD and 51/64 (80%) of STAD but only in 4/75 (5%) of UCEC cases, suggesting that certain tumour types or tumour environments are favourable to the occurrence of particular MSI events. Given the inactivating nature of frameshift MSI events in coding regions, the absence of MSI in known oncogenes such as *BRAF* likely represents the pressures of negative selection in the context of the MSI-H phenotype. Indeed, *BRAF* V600E mutations are observed in 22 of the 45 (49%) MSI-H COAD tumours, but only 4 (2%) frameshift MSI events are observed within the gene.

**Figure 2:**
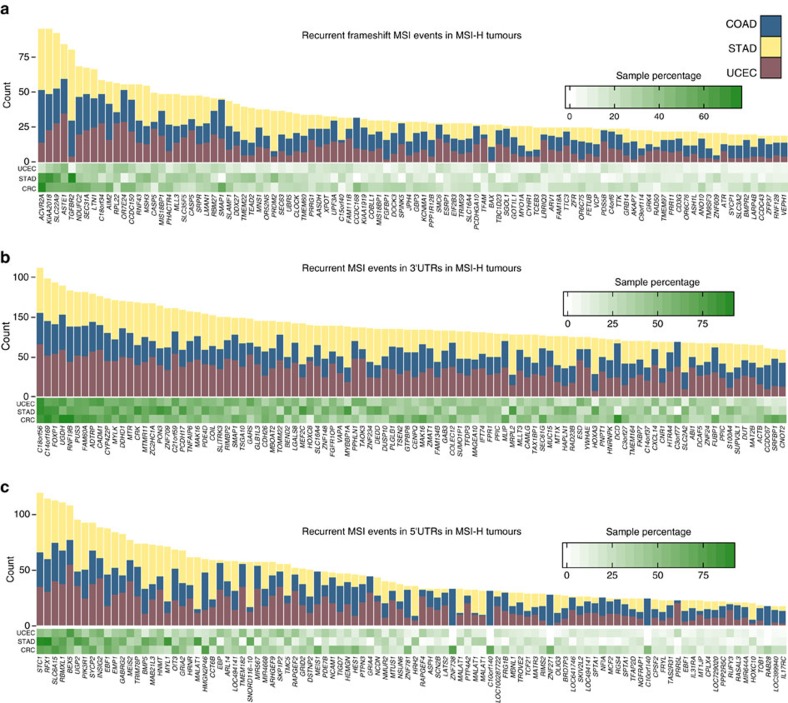
MS loci recurrently altered by MSI. (**a**) Coding MSI loci recurrently targeted by frameshift MSI in CRC (COAD and READ), STAD and UCEC MSI-H tumours. The heatmap shows the fraction of CRC, STAD and UCEC MSI-H tumours containing frameshift MSI events in MS loci located within the coding sequence of the genes indicated on the *x* axis. The total count of frameshift MSI events at these loci is depicted in the above barplot. The full list of MS loci recurrently altered by frameshift MSI is given in Supplementary Data 4. Similarly shown for genes with frequent 3′ UTR (**b**) and 5′ UTR (**c**) MSI events in three MSI-prone tumour types.

To uncover other MS loci frequently targeted by frameshift MSI mutations, we first ranked MS loci by the recurrence level of frameshift MSI events in COAD, STAD and UCEC MSI-H tumours. This analysis resulted in 16,812 frameshift MSI events across a set of 6,441 coding MS loci spanning 4,898 genes (Fig. 2a; Supplementary Data 5). The most recurrent frameshift MSI events are found in *ACVR2A* (51.6% of the tumours), *KIAA2018 (*51%), *SLC22A9* (50%), *ASTE1* (45%), *TGFBR2* (44%), *NDUFC2* (36%), *LTN1* (36%) and *SEC31A* (36%). Frameshift MSI events often display significant tumour-type specificity, for example, *MLL3*, *PRDM2*, *C9orf114*, *BAX* and *OR7E24* are enriched in STAD, *JAK1*, *TFAM* and *SMC6* are enriched in UCEC, whereas *SEC31A*, *C18orf34*, *NDUFC2*, *KIAA1919*, *CCDC168* and others, are enriched in COAD (*P*<0.05, one-tailed Fisher's exact test). Among low-frequency MSI events, *SMAP1*, *CCDC168* and *SPINK5* harbour frameshift mutations in COAD and UCEC but not in STAD tumours.

By analysing the frequency of MSI events in untranslated regions, we found that MS loci within the 3′ UTR region of *C18orf56*, *C14orf169*, *FOXP1*, *UGDH*, *RNF19B*, *PUS3* and *FAM60A* as well as the 5′ UTR region of *STC1*, *RBMXL1*, *RFX1*, *BEX5* and *SLC6A15* are recurrently altered by MSI across MSI-H cases (Fig. 2b,c; Supplementary Data 6 and 7). Other MS loci display marked cancer-type specificity, for example, MSI events within the 5′ UTR region of *ZNF738*, *C10orf140*, *ZNF271* and *RAB28* are specific to COAD tumours, whereas those in *EBP*, *TMEM182*, *MIR567* and *MEIS1* are absent or substantially depleted in STAD compared to COAD and UCEC tumours. Supplementary Data 8 reports the enrichment of frameshift, 3′ and 5′ UTR MSI events in COAD, STAD and UCEC.

To obtain a comprehensive MSI landscape on a pan-cancer scale, we next extended our analysis to all exomes, irrespective of their status as MSI-H, MSI-L or MSS. We observe frameshift MSI events in 8,011 MS loci, of which 51 are altered in more than 50 samples (Supplementary Fig. 7; Supplementary Data 9). *ACVR2A*, *TGFBR2*, *KIAA2018*, *ASTE1* and *SLC22A9* frequently harbour MSI events in STAD and COAD, whereas several other genes are mostly specific to a single tumour type. For instance, *FAM129A*, *GMIP* and *NEK3* are altered in 107 (12%), 93 (10%) and 53 (6%) BRCA (breast cancer) tumours, respectively; *ABT1* and *SLC22A24* are altered in 19.6 and 14% of ACC (adrenocortical carcinoma) tumours; and *ALPK2* and *DPYSL2* are altered by frameshift MSI in 73 (17%) and 59 (14%) OV (ovarian serous cystadenocarcinoma) patients, respectively, but only in 62 of the remaining samples. Although the cancer-related roles of these novel MSI targets are largely unknown, it has been shown that siRNA-mediated inhibition of *ALPK2* inhibits apoptosis^30^, suggesting that the functional implication of these novel, recurrent MSI events warrants further investigation.

### A genome-wide mutational spectrum of MSI

**Figure 3:**
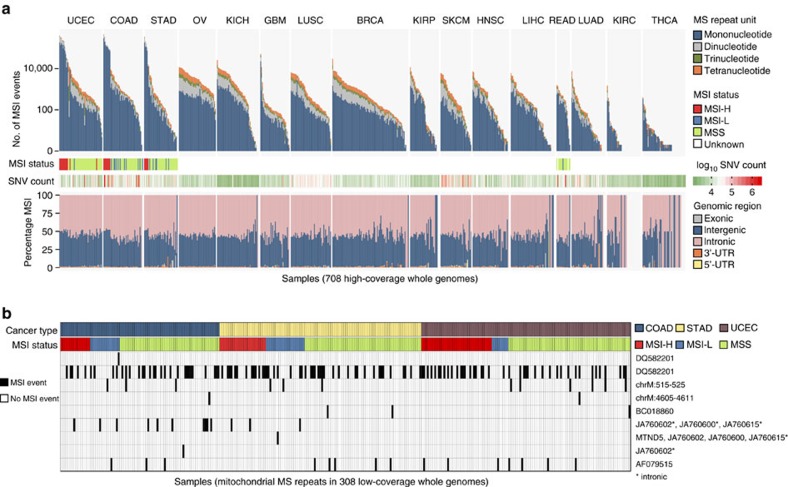
Pan-cancer landscape of genome-wide MSI. (**a**) The first panel shows the number of MSI events across 708 whole genomes, stratified by the length of the repeat unit. The second and third panels report the MSI status and the total count of SNVs, respectively. The fourth panel shows the distribution of MSI events across the genome. (**b**) Landscape of MSI in mitochondrial DNA across 308 COAD, STAD and UCEC low-pass whole genomes. MSI events, including frameshift and in-frame mutations, are shown in black.

Among the non-coding MSI events, those occurring in regulatory elements can function as cancer drivers, similar to somatic SNVs in enhancer regions that have been shown to play a role in tumorigenesis^31^,^32^. To profile the distribution of MSI events genome-wide, we analysed 708 whole-genome (mean coverage: 55 × ) pairs of tumour and matched non-neoplastic samples spanning 16 cancer types (Fig. 3; Supplementary Data 10). The number of MSI events in MSI-H tumours differs significantly from that in MSI-L (*P*=6.25 × 10^−11^, Kolmogorov–Smirnov) and MSS (*P*=4.01 × 10^−15^, Kolmogorov–Smirnov) tumours, whereas the numbers are comparable between MSI-L and MSS cases (*P*=0.17, Kolmogorov–Smirnov). As shown in Fig. 3a, when samples are ordered by decreasing number of MSI events within each tumour type, the decrease is gradual, consistent with a continuous rather than a dichotomous phenotype.

The numbers of exonic MSI calls identified using whole-genome and exome-sequencing data show a high correlation (*r*=0.90, *P*<10^−15^, Pearson correlation for 531 cases profiled by both assays; Supplementary Fig. 8). However, many MSI events are missed in whole-genome data due to their lower coverage, with only 32% of the exome-based calls reproduced on the genomes with our specified thresholds (based on 23 pairs with at least 50 MSI events in exome data). On the other hand, 13% of the exonic MSI events identified in whole-genome data are missed in the exome-based calls, since the target capture regions do not include many exonic MS repeats.

To analyse the influence of read depth on MSI detection sensitivity, we performed subsampling analysis using a sample with a high number of MSI events and coverage (TCGA-AD-A5EJ; tumour at 82 × , matched normal at 42 × ) (Supplementary Figs 9 and 10). We find that the number of MSI events recovered decreases substantially when the coverage is reduced to 20–30 × . However, we do not see a clear relationship between the number of MSI events and coverage above that level of coverage (Supplementary Fig. 10), suggesting that sequencing coverage is not a major factor. We also examine the correlation between the number of MSI events and tumour purity (Supplementary Fig. 11)^33^. The number of MSI events identified in high purity samples spans five orders of magnitude, whereas we systematically detect fewer than a thousand MSI events in the case of low-purity samples. However, the number of low-purity (for example, <0.6) samples in our set is small, meaning that the impact of coverage due to variation in purity does not have a substantial impact on our analysis.

The genome-wide density of MSI events along the chromosomes does not show statistically significant correlation with SNV density, regardless of the bin size (100 kb–10 Mb bins; Supplementary Fig. 12). We have previously used exome sequencing to identify a portion of MSI events in UTRs^15^. With whole-genome data, the number of MSI calls in 3′ UTRs is substantially larger (Supplementary Fig. 8b–d). This allowed us to find that MSI events are enriched in 3′ UTRs for MSI events in 21 out of 25 MSI-H cases (84%), whereas they are depleted in 5′ UTRs and coding regions in 24 out of 25 MSI-H cases. In MSS tumours, only 3 out of 105 (3%) show enrichment of MSI in 3′ UTR regions, whereas 42 (46%) show depletion of MSI events in coding regions (*P*<0.05; one-tailed Fisher's exact test). Overall, these results suggest that MSI events in 3′ UTRs may be under positive selection in MSI-H tumours. The shortening of 3′ UTRs in cancer cells is known to increase the stability of transcripts and thus the translational level of oncogenes^34^. The frequent MSI events in 3′ UTRs may have similar functional consequences (for example, the loss of miRNA-mediated regulation), although they often result in downregulation of their corresponding genes^35^.

We find that 62% of the tumours harboured more than 100 MSI events genome-wide, including samples from all 16 cancer types examined. We again observe a substantial level of intra-tumour type heterogeneity in MSI abundance, with the number of MSI events varying up to 5 orders of magnitude. The presence of non-coding MSI events genome-wide in tumour types beyond the MSI-prone cases is notable. For instance, we observe that 81 and 53% of OV and KICH (kidney chromophobe) samples harbour >1,000 MSI events, exceeding the previously reported MSI-H frequencies of OV (12%) and overall kidney cancers (6%)^36^,^37^. Elevated microsatellite alterations at selected tetranucleotides (EMAST) has been observed in non-MSI-prone tumour types such as lung, head and neck cancers as well as melanoma^38^, but the current markers for EMAST could not be captured with the short reads in our data because of their size.

To investigate the relationship between epigenetic features and the genome-wide distribution of MSI events, we selected the 50 genomes displaying the highest MSI rates and compared their MSI density with the 25-state chromatin state map based on 12 epigenetic marks across 127 epigenomes^39^ (Methods; Supplementary Figs 13–16). For best estimates, the chromatin state map from the most ‘similar' tissue type was used for each tumour type. Our analysis reveals significant enrichment for MSI events in actively-transcribed regions, promoters and enhancers in most MSI-H genomes (two-tailed Fisher's exact test, *P*<0.05; Supplementary Fig. 17). On the other hand, inactive regions, including constitutive heterochromatin, repressed Polycomb, bivalent promoters and quiescent chromatin, are significantly depleted for MSI overall. Taken together, these results show the over-representation of MSI in functionally important, typically open-chromatin regions, extending our previous results based on seven colorectal and UCEC tumours^15^.

### MSI events in the mitochondrial genome

To obtain a mitochondrial MSI landscape, we analysed TCGA low-coverage (6–8 × ) whole-genome data, since the number of low-coverage samples of the MSI-prone tumour types was larger than the number of high-coverage samples of the same tumour types. Due to their high copy number, the mitochondrial DNA had a median coverage of >800 × even in the low-coverage data^40^. We applied our MSI discovery pipeline to a set of 31 mitochondrial MS loci (Supplementary Data 11) across 308 cancer genomes (COAD, STAD and UCEC) (Fig. 3b; Supplementary Data 12). The most recurrent MSI event is observed in *DQ582201*, a polyC mononucleotide repeat (115 MSI events, 37% of tumours); the second most recurrent event is on the exon regions of *AF079515* (15 MSI events, 5% of tumours). The instability of *DQ582201* has been reported in several cancer types^41^. The majority of mitochondrial MS loci (22 out of 31 MS loci; 71%), however, do not contain MSI events in any of the tumours examined, suggesting that mitochondrial MSI is not widespread compared to nuclear MSI. Moreover, mitochondrial MSI events are not associated with the MSI status of the tumour: 43 (54%), 22 (48%) and 98 (54%) mitochondrial MSI events are observed in MSI-H, MSI-L and MSS genomes examined, respectively. The relationship between the nuclear MSI and mitochondrial MSI has been controversial^41^, and our mitochondrial genome-wide MSI examination suggests that these two events are not correlated.

### A panel of MS loci frequently mutated in MSI-H cases

**Figure 4:**
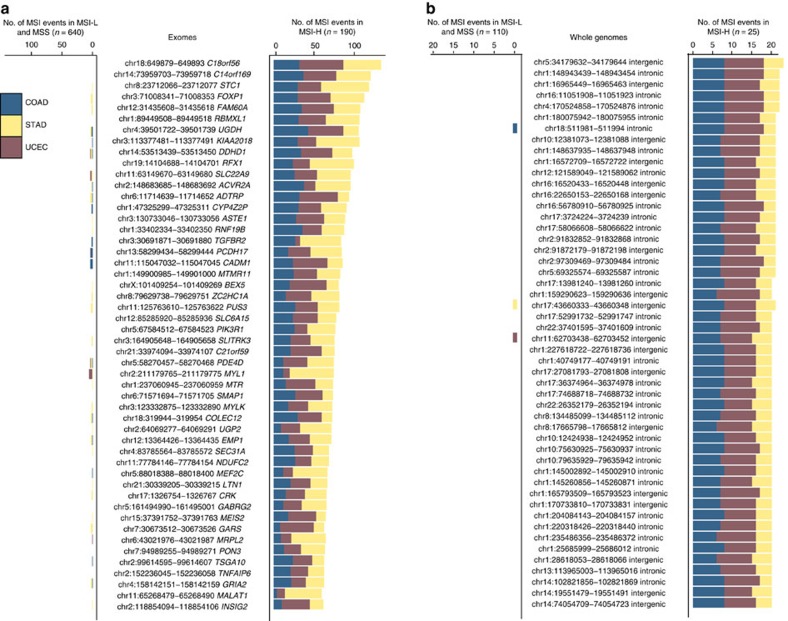
MS repeats recurrently altered by MSI in MSI-H tumours. (**a**) The barplots report the number of COAD, STAD and UCEC tumours harbouring MSI events at the loci indicated in the central panel. This analysis examined 190 MSI-H, 118 MSI-L and 522 MSS exomes. (**b**) The recurrence analysis was extended to 25 MSI-H, 19 MSI-L and 105 MSS whole genomes. Genomic coordinates in **a**,**b** indicate the location of the MSI repeats in the hg19 assembly of the human genome.

We focused on frameshift MSI events earlier to understand the functional impact of coding MSI in MSI-prone tumours. Here, we sought to uncover mutational patterns discriminative of MSI-H status across exonic and non-coding MS loci, as well as to identify hyper-mutable MS loci across the entire genome. We first ranked recurrently targeted MS loci by their specificity for MSI-H tumours using exome-sequencing data from COAD, STAD and UCEC tumours. Our analysis yielded a catalogue of MS loci specific to MSI-H tumours (Fig. 4a; Supplementary Data 13). We find that several of these MS loci lie within genes prone to frameshift MSI events, such as *KIAA2018*, *ACVR2A* or *ASTE1* (ref. 15). In contrast to frameshift and 3′/5′ UTR MSI events (Fig. 2), few MSI events enriched in MSI-H and depleted in MSS/MSI-L cases display cancer-type specificity, implying that there are commonalities in the molecular mechanisms underlying MSI at these loci across the three cancer types.

Given that most MS loci lie within the non-coding genome, we also extended our recurrence analysis to whole genomes by utilizing sequencing data from 25 MSI-H, 19 MSI-L and 105 MSS tumours. We discovered a set of intronic and intergenic MS repeats recurrently targeted by MSI in MSI-H cases (Fig. 4b; Supplementary Data 14). Perhaps not surprisingly given the larger list of candidate MS, these non-coding loci are more specific to MSI-H than the best exonic loci are (*cf.* Fig. 4a), containing MSI events in nearly all of the MSI-H tumours and almost none in MSI-L or MSS samples. These inquiries have yielded a collection of coding and non-coding MS loci recurrently targeted by MSI in MSI-H tumours, which provide a foundation to refine and extend the set of markers employed for MSI-H categorization.

### Prediction of MSI status from exome-sequencing data

**Figure 5:**
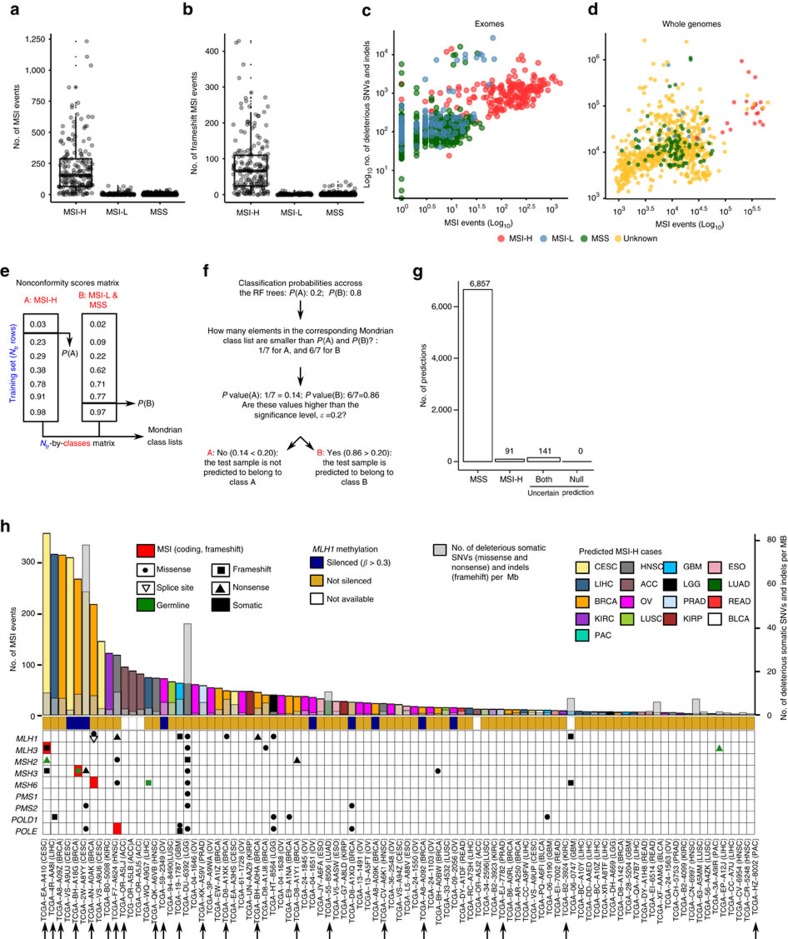
Distribution of the number of MSI and prediction of MSI status. Distribution of the number of MSI (**a**) and frameshift MSI events (**b**) in MSI-H and MSS (also including MSI-L) tumours. Correlation between the number of SNV and MSI events in exomes (**c**) and whole genomes (**d**). Prediction of MSI status from exome-sequencing data using conformal prediction and random forest models (**e**). Initially, we used 10-fold cross-validation to calculate predictions for all training examples. The fraction of trees in the forest voting for each class was recorded, and subsequently sorted in increasing order to define one Mondrian class list per category. (**f**) The model which was trained on all training data was applied to 7,089 exomes. For each of these samples, the algorithm recorded the fraction of trees voting for each class. The *P* value for each class was calculated as the number of elements in the corresponding Mondrian class list higher than the vote for that class (for example, 6 out of 7 in the toy example depicted in Fig. 5f) divided by the number of elements in that list. If the *P* value for a given class is above the significance, *ɛ*, the sample is predicted to belong to that category. The confidence level (1−*ɛ*) indicates the minimum fraction of predictions that are correct. (**g**) Number of samples predicted as MSI-H, MSS and uncertain (both: cases in which the classifier does not have enough power to confidently assign a single category; none: cases in which when the samples that are outside the applicability domain of the model). Here, the confidence level was set to 0.75. (**h**) Landscape of MSI for the 91 exomes predicted as MSI-H at a confidence level of 0.75. Samples predicted to be MSI-H at a confidence level of 0.80 are marked with black arrows.

The total numbers of MSI and frameshift MSI events are significantly higher in MSI-H tumours than in MSI-L or MSS tumours (*P*<10^−15^; Kolmogorov–Smirnov test; Fig. 5a,b). The number of SNV and MSI events exhibit moderate to low correlation in MSI-H (*r*=0.32, *P*=6.15 × 10^−6^ in exomes, and *r*=0.35, *P*=0.09 in whole genomes; Pearson correlation; Fig. 5c,d), MSI-L (*r*=0.10, *P*=0.68, Pearson correlation) and MSS (*r*=−0.06, *P*=0.56, Pearson correlation) tumours. To test whether our MSI calls can be used to distinguish between MSI-H and MSS cases, we built random forest^42^ classification models. Each tumour was encoded by a vector recording the presence or absence of MSI events across MS loci, as well as the total number of MSI events (Methods). Models built on a limited set of learning examples (that is, only those 190 MS-H, 118 MSI-L and 533 MSS tumours with MSI status annotations) are likely to possess limited predictive power on external data. Thus, we included conformal prediction^43^ in our modelling pipeline to provide confidence estimates for individual predictions (Fig. 5e,f; Methods). Briefly, conformal prediction evaluates the similarity (that is, conformance) between the new samples and the training data. The output represents the probability that the new sample is either MSI-H, MSS or uncertain (in the case of the new samples being outside the applicability domain of the model), given a user-defined significance level that sets the maximum allowable fraction of erroneous predictions. Our 10-fold cross-validation (CV) showed high accuracy of the models produced (sensitivity: 92%; specificity: 99%). Comparable results were obtained in leave-one-out CV (sensitivity: 93%; specificity: 99%), indicating that the MSI events detected using whole-exome data convey enough predictive signal for MSI categorization.

By applying the prediction model to 7,089 exomes from 17 cancer types not commonly tested for MSI status, we identified 91 additional MSI-H cases using a confidence level of 0.75, 22 of which were identified at confidence level of 0.80 (Fig. 5g,h; Supplementary Data 15). Among the 91 cases, the most frequent are BRCA (16), OV (14) and LIHC (liver hepatocellular carcinoma; 11). Our estimated MSI-H rate for OV is 3.2%, significantly lower than that reported previously (10%)^44^; for HNSC (head and neck squamous cell carcinoma) and CESC (cervical cancer), our estimated MSI-H rates are 1.2% and 2.3%, whereas the reported rates in the literature are 3% and 7% (ref. 8). The frequencies generated for the other non-MSI-prone cancer types were mostly in agreement with the reported numbers in the literature^8^. For example, our estimated MSI-H frequencies for PRAD (prostate adenocarcinoma), LUAD (lung adenocarcinoma) and LUSC (lung squamous cell carcinoma) are 0.6, 0.2 and 1.2%, respectively, which are comparable to the frequencies of 1% and 0–2% reported for prostate and for lung cancers, respectively^8^. We note that the differences in the rates may be due to the small sample sizes used in the literature for some tumour types^8^, differences in the characteristics of the cohorts (for example, tumour stage) and tumour-type-specific features that were missed in our model. We did not identify any MSI-H cases among THCA (papillary thyroid carcinoma; *n*=493), PHCA (pheochromocytoma; *n*=179) and SKCM (skin cutaneous melanoma; *n*=109) tumours. Overall, the frequency of MSI-H cases in non-MSI-prone cancer types was found to be 1.3%, significantly lower than the 14% we observed in UCEC, STAD, COAD, READ and ESCA tumours. Consistent with our analyses of COAD, READ, STAD, ESCA and UCEC MSI-H tumours (Fig. 1b), we found that the number of MSI events varied markedly across these newly identified MSI-H tumours (Fig. 5h). We detected 1,365 frameshift MSI events in the tumours predicted as MSI-H, with the most frequent incidences in *DPYSL2* (12 cases), *OR11G2* (9), *SLC22A9* (9) and *KIAA2018* (8), suggesting that the MSI events that recur in MSI-H cases (*cf.* Fig. 2) constitute a mutational signature that is leveraged by the predictive model for MSI categorization. We find that 31 patients display somatic mutations in MMR genes, and 1 CESC (TCGA−EA−A410) and 2 LIHC (TCGA-WQ-A9G7 and TCGA-EP-A12J) cases harbour germline mutations in *MSH2*, *MSH6* and *MLH3*, respectively. In addition, we observe that 1 BRCA patient (TCGA-BH-A18G) harbours a missense germline mutation predicted to be pathogenic with high confidence (Methods) and a somatic frameshift event in *MSH3*.

We also performed mutation signature analysis based on the mutation frequencies of 96 trinucleotide contexts^45^ (Supplementary Fig. 18). For the 91 MSI-H predicted cases, we confirm the mutation signatures characteristic of MSI-H cases, for example, C>T transitions in (A/C/G)pCpN sequence contexts and C>A transversions at an CpCpN context, suggesting that the mutation signatures of predicted MSI-H cases are largely concordant with those of known MSI-H cases.

## Discussion

Our joint analysis of MSI-H tumours from multiple cancer types has revealed that several DNA repair pathways other than MMR, including ATR, BER, HR and NHEJ, are altered by single-nucleotide and MS mutations. Moreover, we have uncovered new genes affected by frameshift MSI events in MSI-prone tumours as well as in tumour types not frequently affected by MSI (for example, *FAM129A*, *GMIP* and *NEK3* in BRCA, and *DPYSL2* and *ALPK2* in OV). Some of these genes have shown strong predictive power for MSI-H status (for example, *ACVR2A* and *KIAA2018* for COAD, STAD and UCEC), whereas others display low recurrence for single cancer types (for example, *SMAP1* for STAD). Along with the diverse molecular functions enriched for MSI events in these tumour types, our data reaffirm that some genes are particularly susceptible to MSI in specific cancer types^46^. Although some of their potential cancer-related roles have been identified^47^, the functional relationship between MSI and tumorigenesis as well as the similarity of molecular mechanisms that establish the MSI phenotype across cancer types remain to be validated.

By classifying 7,089 patients into MSI-H and MSS categories using our MSI-based predictive model, we identified 91 new MSI-H cases from 16 different tumour types. According to our classification model, the frequency of MSI-H cases in MSI-prone tumours is roughly ten times larger than in other tumour types (14.5% versus 1.3%). In contrast to previous models based on SNVs or mononucleotide repeats^48^,^49^, our modelling approach estimates the likelihood of prediction error for individual patients using a confidence level, which can be easily interpreted (for example, a confidence level of 0.9 means that no less than 90% of the predictions should be correct).

Although the search space considered in our whole-genome and exome MSI reference sets is large (∼19 million and 386,396 MS loci, respectively), it comprises only MS repeats of size 6–60 bp and up to tetrameric repeats. Although this MSI calling pipeline captures the vast majority of MS loci (for example, >99% of repeats in our reference MS are smaller than 40 bp)^15^, MSI events in certain non-coding MS loci might have missed the significance threshold due to low coverage, and we anticipate that the rates of MSI events presented here are likely to be under-estimates of the true rates. The Illumina sequencing data used in this study are sufficiently robust for estimating the length of homopolymer runs^50^, but a platform with longer reads will help in a more comprehensive identification of MSI target loci. A further increase in the number of samples annotated with MSI status will also increase the power to detect all relevant loci.

Of clinical relevance, we provide the largest catalogue available to date of coding and non-coding MS loci frequently altered across human cancers. As the use of high-coverage (for example, >1,000 × ) gene panels is becoming more common in the clinic, the loci identified in our study, especially those in the non-coding regions, can be profiled to serve as highly sensitive markers for MSI across multiple tumour types. This will avoid a separate test for MSI in MSI-prone tumours, and it will identify the small set of MSI patients in non-MSI-prone tumours in which MSI is rarely considered by clinicians. The potential benefit of such a panel is enormous, given the demonstrated efficacy of immunotherapy for the MSI cohort^13^.

## Methods

### Data sets

Exome and whole-genome tumour-normal pairs from the TCGA project were downloaded from CGHub (http://cghub.ucsc.edu). The reads were mostly 100 bp paired-end reads and were aligned to the NCBI build 37 (hg19) using BWA. The full list of samples is given in Supplementary Data 1, 10 and 12. The MSI status (MSI-H, MSI-L and MSS) were downloaded from the GDAC (https://gdac.broadinstitute.org) website, whereas the methylation state of the *MLH1* promoter, gene expression and DNA copy number variation data were downloaded from the Genomics Data Commons Data Portal (https://gdc-portal.nci.nih.gov/) websites. MSI status was evaluated by the TCGA consortium for COAD, READ, ESCA, STAD and UCEC tumours using a panel of four mononucleotide repeats (BAT25, BAT26, BAT40 and TGFBRII) and three dinucleotide repeats (D2S123, D5S346 and D17S250), except for a subset of COAD/READ genomes evaluated by five mononucleotide markers (BAT25, BAT26, NR21, NR24 and MONO27)^51^. Tumours were classified as MSI-H (≥40% of markers altered), MSI-L (<40% of markers altered) and MSS (no marker altered).

### Defining a reference set of MS repeats

To generate an exome-wide reference set of MS loci, we utilized the Sputnik algorithm^15^ to identify MS loci in the mRNA sequences of 39,496 RefSeq genes (USCS Genome Browser, hg19). We limited our analysis to mono-, di-, tri- and tetranucleotide MS loci of size 6–60 bp, which can be detected reliably with enough flanking sequences from 100 bp reads. We derived the reference set of MS repeats from RefSeq sequences as the target regions used by the TCGA are different across cancer types. MS repeats falling within splice sites were removed, as they have undetermined genomic coordinates or are redundant to multiple isoforms. The final reference set of exonic MS sites comprised 386,396 loci (112,896 mono-, 63,162 di-, 132,117 tri- and 78,221 tetranucleotides). These included 154,590 coding, 50,598 5′-UTR and 181,208 3′-UTR MS loci, as annotated in the UCSC Genome Browser.

To produce a genome-wide reference set of MS loci, a total of 19,039,443 MS repeats were obtained using the Sputnik algorithm (chromosome 1 through Y) and categorized into five groups (coding, 248,100; 5′-UTR, 39,582; 3′-UTR, 166,111; intronic, 8,265,436; intergenic, 10,320,214). This MS set encompasses 7,404,614 mono-, 3,686,129, di-, 3,750,887 tri- and 4,197,813 tetranucleotides. We also utilized the Sputnik algorithm to build a reference set of mitochondrial MS loci from the hg19 mitochondrial DNA (mtDNA), which contained a total of 31 MS loci (10 mono-, 2 di-, 11 tri- and 8 tetranucleotides) (Supplementary Data 11).

### Detection of DNA slippage events

After filtering reads with low mapping quality, intra-read MS repeats were identified with the same method used to identify reference MS repeats and were intersected with the reference MS repeats. We note that the minimum size of intra-read repeats detected was 5 bp. Thus, reads spanning MS repeats contracted below 5 bp were not considered. We required the 2 bp flanking sequences (both 5′ and 3′) of the intra-read MS repeats to be identical to those of matching reference repeats, thereby discounting truncated MS repeats. In each genome, the distribution of the allelic repeat length at each MS locus was obtained by collecting the lengths of all intra-read MS repeats mapped to that locus. We compared the distributions of MS lengths from tumour and matched normal genomes at each locus using the Kolmogorov–Smirnov statistic. An FDR of <0.05 was used as a threshold for statistical significance, with a minimum of 5 tumour and 5 matched normal reads. We note that the number of MSI ‘events' refers to the absolute number of MSI counts per sample, whereas sample percentage refers to the percentage of samples from a given cancer-type harbouring MSI events at a particular MS locus. We distinguished MSI events at coding sequences into in-frame and frameshift events depending on whether the difference between (i) the mode of the read length distribution of the normal samples and (ii) the mode of the read length distribution of the tumour sample or the second most frequent read length from this distribution (if supported by at least 20% of the reads) was a multiple of three.

### Mutation calling

We utilized MuTect 1.1.4 (ref. 52) to call somatic mutations in both the tumour and matched normal whole-genome samples, using the Catalogue of Somatic Mutations in Cancer (COSMIC) v68 and dbSNP135 as reference sets of known somatic and germline mutations, respectively. To ensure the somatic origin of the variant sets reported by MuTect, we filtered out germline mutations from the 1000 Genomes Project (phase 3, release 20130502)^53^ and any mutation present in at least one read in two unmatched normal BAM files from the same tissue. Somatic mutations for all 7,919 exomes were downloaded from the GDAC (https://gdac.broadinstitute.org) website. We utilized HaplotypeCaller 3.4-46-gbc02625 (ref. 54) to examine germline mutations. We only kept deleterious mutations (that is, frameshift, nonsense, missense and splicing site) supported by at least 10 reads, and those with at least 30% of the reads mapped to that locus supporting the alternative allele. In addition, we only kept missense mutations with a predicted MetaLR score from Annovar^55^ higher than 0.9. We did not consider mutations in the exons 9, and 11 to 15 of *PMS2*, as the *PMS2CL* pseudogene displays more than 98% sequence identity with these exons. Due to the high allelic diversity of *PMS2CL* due to sequence transfer^23^, it is challenging to dismiss false positive mutations called in these exons.

### Correlation between gene expression and MMR alterations

To investigate the association between the level of gene expression and genomic events on seven MMR genes (*MLH1*, *MLH3*, *MSH2*, *MSH3*, *MSH6*, *PMS1* and *PMS2*) and two proofreading DNA polymerases (*POLD1* and *POLE*), we utilized gene expression, promoter methylation and DNA copy number profiles for the 186 MSI-H cases with available data from these three data types.

Gene expression profiles were first log transformed, that is; log_2_ (FPKM+1). Subsequently, the expression values of each row and column were median-centered and rescaled so that the sum of the squares of the values are 1. To process the promoter methylation data, we collected 17 common probes corresponding to the nine genes studied between two microarray platforms (humanmethylation27 and humanmethylation450, Illumina). *β* values were obtained for 17 probes and averaged per gene. The *MLH1* promoter was considered methylated in samples with *β* values>0.3. To obtain copy number data, we selected segmentation files filtered for germline alterations. Log2 copy numbers overlapping the genomic segments of eight genes were considered as the copy numbers of these genes. *POLE* was ignored since it was not covered by the segmentation files. Pearson's correlation was used to assess the relationship between gene expression and promoter methylation (*β* values), as well as the relationship between gene expression and DNA copy numbers. The relationship between gene expression and somatic mutations and MSI events, was evaluated using the Mann–Whitney test (*α*=0.05).

### Analysis of epigenomic features

We downloaded the coordinates of the 25-state chromatin state map defined using 12 marks (H3K4me1, H3K4me2, H3K4me3, H3K9ac, H3K27ac, H4K20me1, H3K79me2, H3K36me3, H3K9me3, H3K27me3, H2A.Z and DNase) across 127 reference epigenomes from the Epigenome Roadmap project^56^. For each of the 30 whole genomes with the highest MSI counts, the list of their MSI loci was intersected with the chromatin state maps defined using cell lines from the same anatomical location as the tumour types. We used the chromatin state maps defined using the epigenomes E092, E094, E0110 and E0111 for STAD, E117 for UCEC, E076, E106 and E075 for COAD, E086 for KICH, E027, E028 and E199 for BRCA, E053, E054, E067, E068, E069, E070, E071, E072, E073, E074, E081, E082 and E0125 for GBM, E097 for OV, E088, E096, E114 and E128 for LUSC, E055, E056, E057, E059, E061, E126, E127 and E058 for HNSC and E086 for KIRP. Subsequently, the percentage of MSI events overlapping each chromatin state was averaged across the matched epigenomes. The same process was applied to the set of MS loci from the genome-wide reference set. Fisher's exact test was used to assess the significance of the enrichment for MSI events of each chromatin state in each of the cancer genomes. The significance level was set to 0.05.

### MSI status prediction

We used random forest models^42^ to build binary classifiers for the prediction of MSI status. Each tumour was encoded with a vector recording the number of MSI events and the presence or absence of MSI events in 7,863 genes targeted by MSI in at least one sample. Features displaying a variance close to zero across all learning examples (that is, near-zero variance descriptors) were removed using the function nearZeroVar from the R package caret^57^. The remaining descriptors were mean-centered to zero and scaled to unit variance to obtain *z*-scores using the function PreProcess from the same package. The number of trees was set to 100 (ref. 43), the optimal value of the parameter mtry was determined to be 182 via 10-fold cross-validation and the default values were used for the remaining parameters. With this mtry value, the final prediction models were built using all available learning examples.

To estimate prediction errors, we used the following pipeline^43^ from the R package conformal (https://cran.r-project.org/web/packages/conformal/index.html). We used cross-validation predictions to define a Mondrian class list for each category (that is, MSI-H and MSS) by sorting in increasing order the fraction of trees voting for that class for each training example. Next, we applied the model trained on all learning examples to each sample without MSI categorization, and calculated for each case the fraction of trees in the forest voting for each class. These values were intersected with the corresponding Mondrian class list. For each sample, the *P* value for a given class was calculated as the number of elements in the corresponding Mondrian class list higher than the vote fraction for that class divided by the number of elements in that list. If the *P* value for a given class is above the significance level, *ɛ*, the sample is predicted to belong to that category. Hence, a given sample may be called as MSI-H or MSS. However, it can also be called as both in cases when the model does not have enough predictive power to discriminate between classes, or neither in cases when the sample is outside the applicability domain of the model. This flexibility thus gives an unbiased estimate of the reliability of the predictions given the training data. The significance level, *ɛ*, indicates the maximum fraction of predictions that are incorrect. Therefore, increasing the confidence level might increase the number of uncertain predictions, that is, samples classified as both MSI-H and MSS.

### Code availability

The code for calling MSI events is available from the authors upon request.

### Data availability

The results published here are based on data generated by The Cancer Genome Atlas and obtained from the Database of Genotypes and Phenotypes (dbGaP) with accession number phs000178.v8.p7. Information about TCGA can be found at http://cancergenome.nih.gov. All other remaining data are available within the article and Supplementary Data, or available from the authors upon request.

## Additional information

**How to cite this article:** Cortes-Ciriano, I. *et al*. A molecular portrait of microsatellite instability across multiple cancers. *Nat. Commun.* **8,** 15180 doi: 10.1038/ncomms15180 (2017).

**Publisher's note**: Springer Nature remains neutral with regard to jurisdictional claims in published maps and institutional affiliations.

## Supplementary Material

Supplementary Information Supplementary Figures

Supplementary Data 1 The TCGA barcodes and the total number of MSI calls showing significant differences (FDR < 0.05; Kolmogorov-Smirnov test) in the MS allele lengths between the tumor and matched normal exomes are listed.

Supplementary Data 2 Results of the DAVID analysis

Supplementary Data 3 Frameshift MSI events observed in MSI-H tumors among 13 out the 151 DNA repair genes examined are listed.

Supplementary Data 4 Frameshift MSI events observed in MSI-H tumors among 12 out of the 130 major cancer-related genes examined are listed.

Supplementary Data 5 The coding MS loci harboring recurrent frameshift MSI events in COAD, STAD and UCEC MSI-H tumors found using exome sequencing data are listed.

Supplementary Data 6 Recurrent MSI events in 3'-UTR regions are listed.

Supplementary Data 7 Recurrent MSI events in 5'-UTR regions are listed.

Supplementary Data 8 Enrichment for frameshift, 3' UTR and 5' UTR MSI events in COAD, UCEC and STAD. The significance was calculated using the one-tailed exact Fisher's text (± = 0.05).

Supplementary Data 9 The coding MS repeats harboring recurrent frameshift MSI events in MSS, MSI-L and MSI-H genomes identified using whole exome sequence data from 22 cancer types are listed.

Supplementary Data 10 The barcodes, the total number of significant MSI calls (FDR < 0.05; Kolmogorov-Smirnov test), and the total number of MS loci profiled are listed for the 708 whole-genomes analyzed.

Supplementary Data 11 List of MS loci discovered in the human mitochondrial DNA.

Supplementary Data 12 The barcodes corresponding to the samples used in the analysis of MSI in mitochondrial DNA are listed.

Supplementary Data 13 Top 1,000 most recurrently altered MS loci in COAD, STAD and UCEC MSI-H exomes.

Supplementary Data 14 Top 1,000 most recurrently altered MS loci in COAD, STAD and UCEC MSI-H whole genomes.

Supplementary Data 15 Predicted MSI status for 7,096 exomes.

## Figures and Tables

**Table 1:** Tumour samples utilized to profile MSI

**Tumour type**	**Abbreviation**	**Samples**	**MSI-Hs (frequency)**
Uterine corpus endometrial carcinoma	UCEC	265	75 (28.3%)
Stomach adenocarcinoma	STAD	292	64 (21.9%)
Colon adenocarcinoma	COAD	271	45 (16.6%)
Rectal adenocarcinoma	READ	76	3; 4* (9.2%)
Adrenal cortical carcinoma	ACC	92	5* (5.4%)
Oesophageal carcinoma	ESCA	183	3; 3* (3.3%)
Ovarian cancer	OV	436	14* (3.2%)
Liver hepatocellular carcinoma	LIHC	375	11* (2.9%)
Cervical squamous cell carcinoma	CESC	305	7* (2.3%)
Breast cancer	BRCA	922	16* (1.7%)
Glioblastoma multiforme	GBM	316	4* (1,3%)
Head and neck squamous cell carcinoma	HNSC	505	6* (1.2%)
Lung squamous cell carcinoma	LUSC	407	5* (1.2%)
Kidney renal clear cell carcinoma	KIRC	377	4* (1.1%)
Pancreatic cancer	PAC	171	2* (1.1%)
Urothelial bladder cancer	BLCA	368	2* (0.8%)
Papillary kidney carcinoma	KIRP	286	2* (0.7%)
Low grade glioma	LGG	514	3* (0.6%)
Prostate adenocarcinoma	PRAD	497	3* (0.6%)
Lung adenocarcinoma	LUAD	482	1* (0.2%)
Cutaneous melanoma	SKCM	109	0* (0%)
Pheochromocytoma and paraganglioma	PHCA	176	0* (0%)
Thyroid cancer	THCA	493	0* (0%)
Total		7,919	281

The Abbreviation column indicates the cancer type abbreviations used throughout the manuscript. The number of cases predicted as MSI-H at a confidence level of 0.75 is indicated with `*' (see subsection `Prediction of MSI status from exome-sequencing data').
