# Supplementary material for: A molecular portrait of microsatellite instability across multiple cancers
Source: Nat Commun. 2017 Jun 6;8:15180. doi: 10.1038/ncomms15180 (PMC5467167; doi:10.1038/ncomms15180)
Supplement: Supplementary Information — Supplementary Figures [file ncomms15180-s1.pdf]

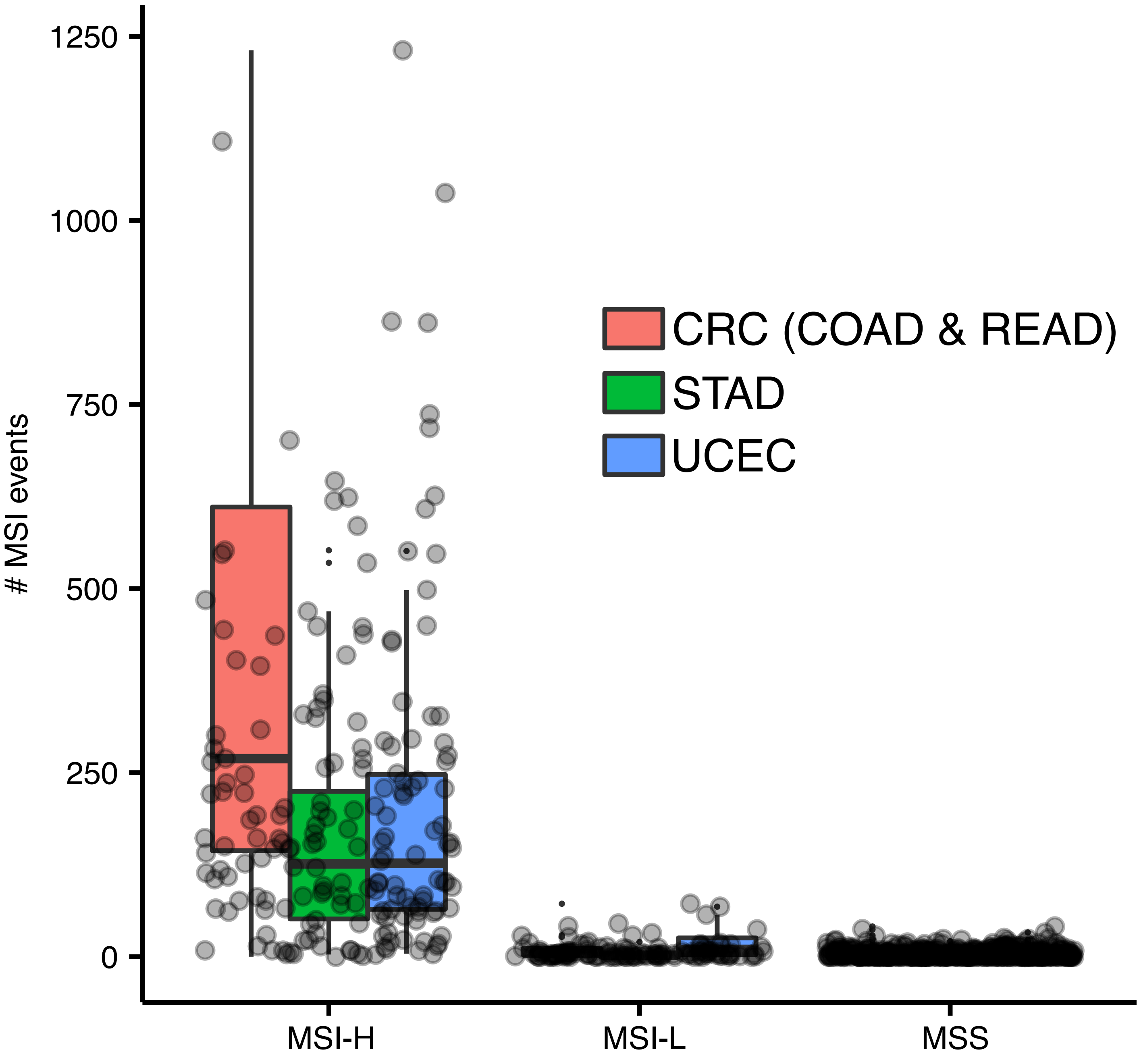


Supplementary Figure 1: Distribution of MSI events in MSI-H, MSI-L and MSS cases in MSI-prone tumor types.

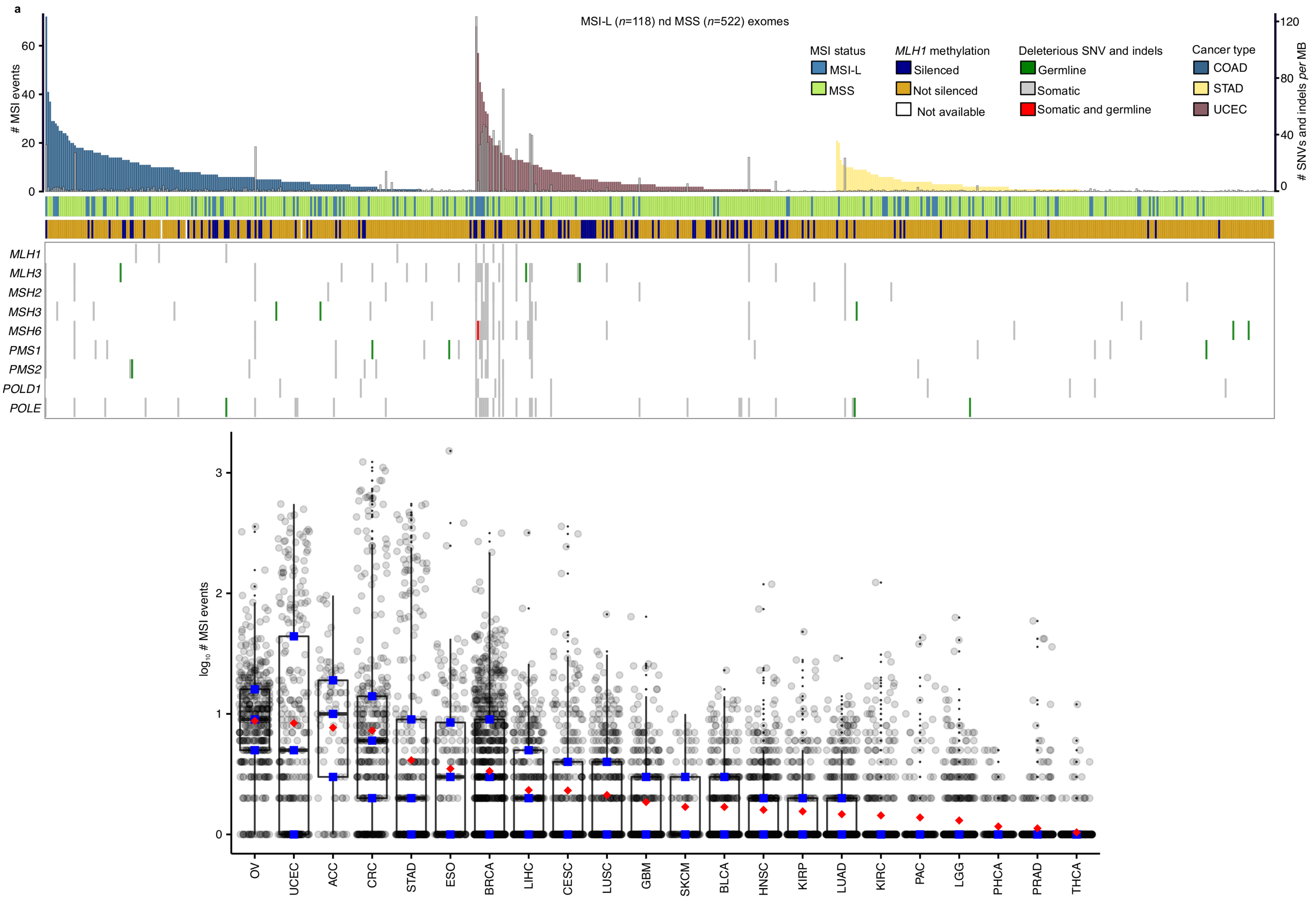


Supplementary Figure 2: (a) Landscape of MSI in MSI-L and MSS tumors. (b) Distribution of MSI events across 22 tumor types. Blue points indicate the median and the interquartile range (25th through 75th percentile), whereas red points indicate the mean value.

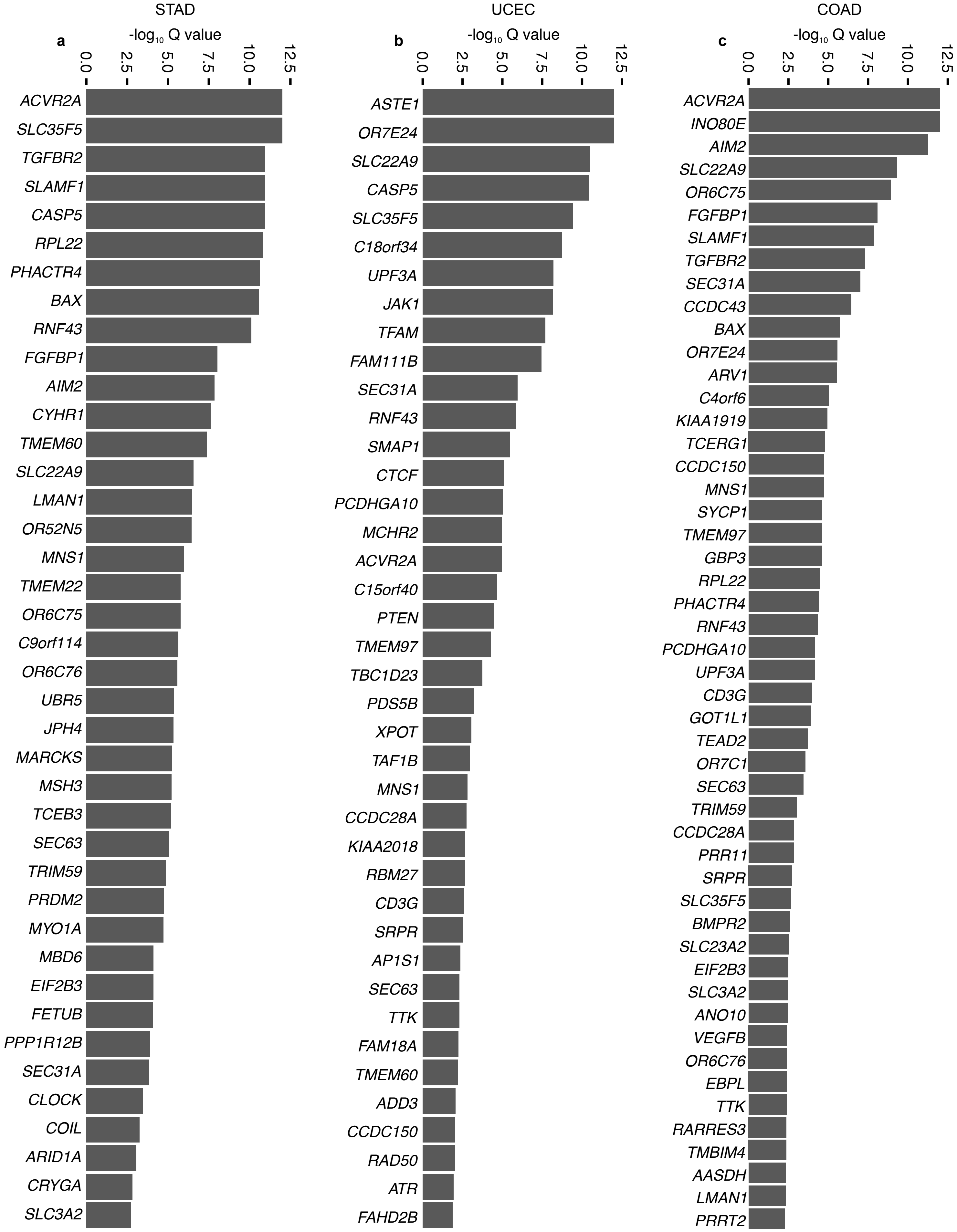


Supplementary Figure 3: Top-ranked genes with significantly recurrent MSI in STAD (a), UCEC (b) and COAD (c) tumors (Q value, FDR < 0.05; MutSigCV).

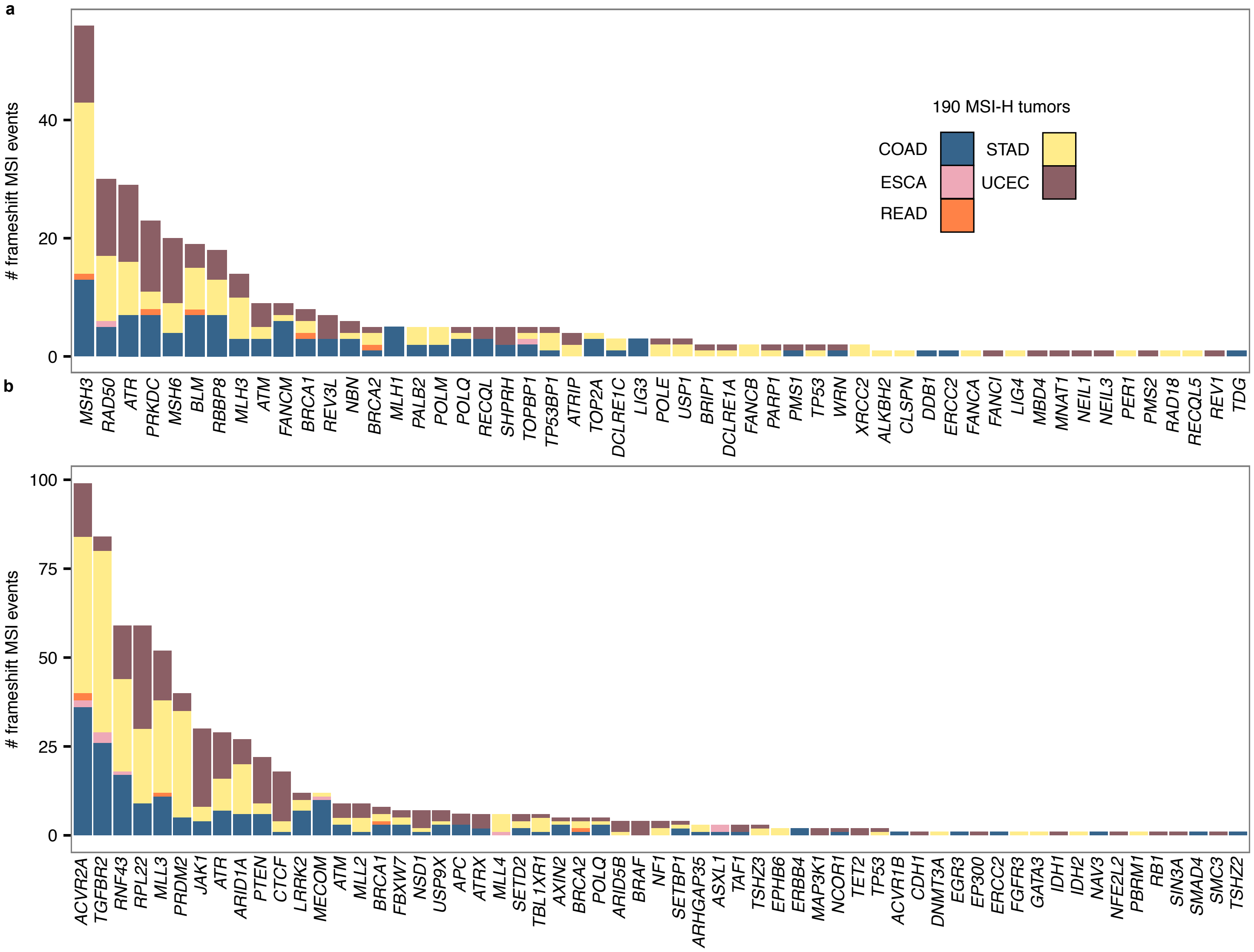


Supplementary Figure 4: Abundance of frameshift MSI events in genes implicated in DNA repair (a) and tumorigenesis (b) across 5 MSI-prone cancer types.

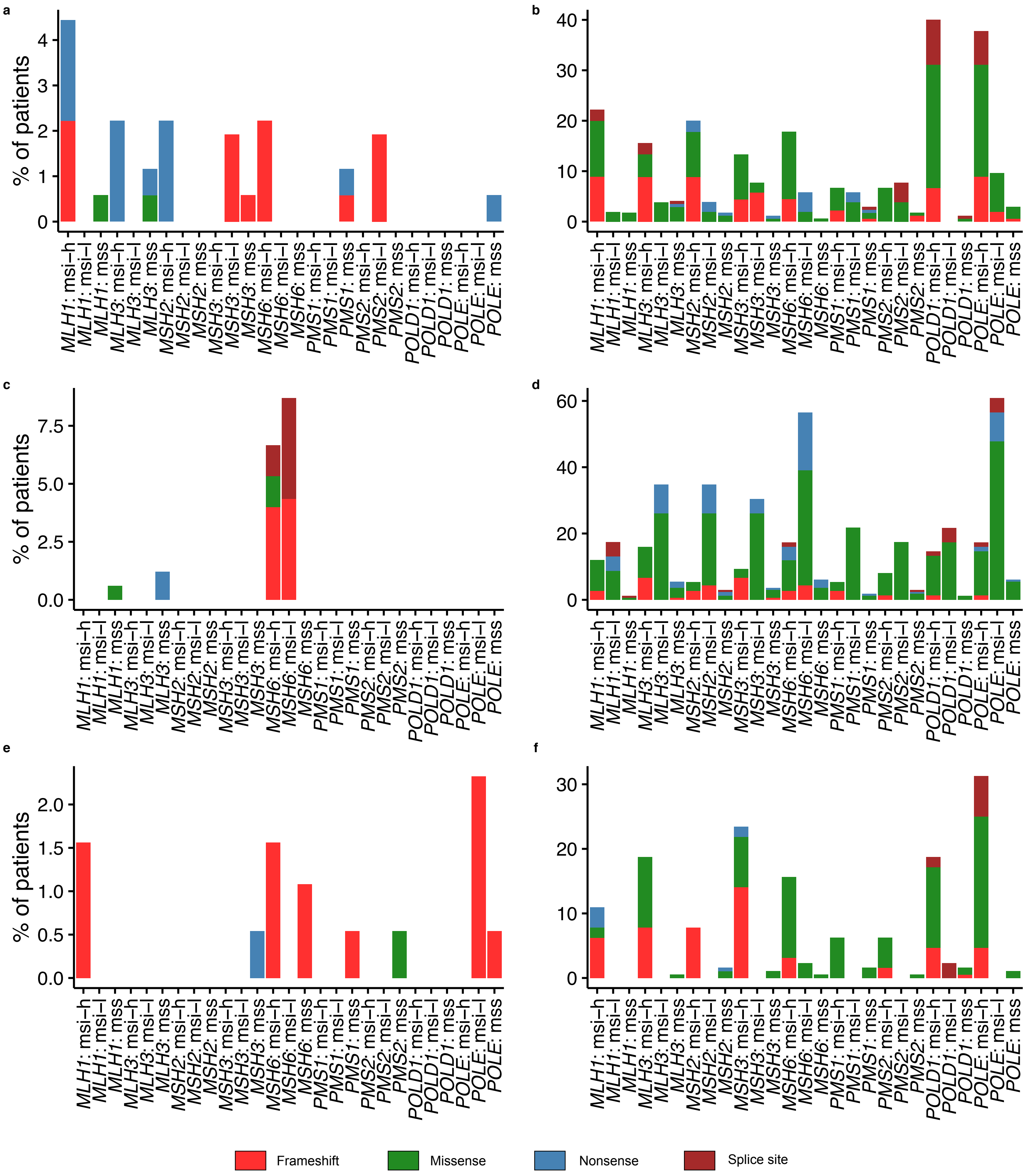


Supplementary Figure 5: Frequency of SNVs and indels in MMR genes, POLE and POLD1 in COAD (a,b), UCEC (c,d) and STAD (e,f).

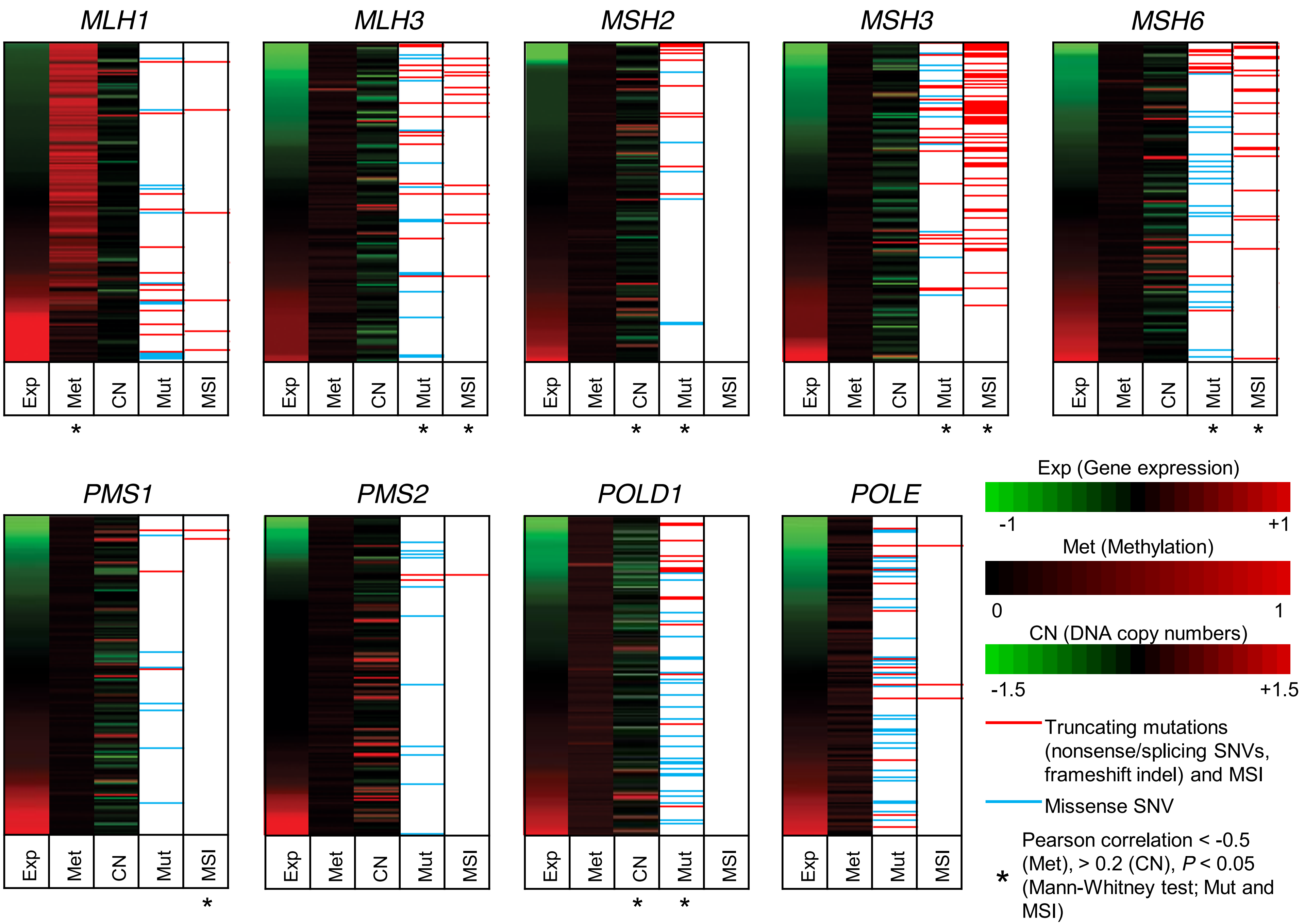


Supplementary Figure 6: Gene expression, promoter methylation and DNA copy number profiles for MMR genes and two proofreading DNA polymerase corresponding to186 MSI-H cases.

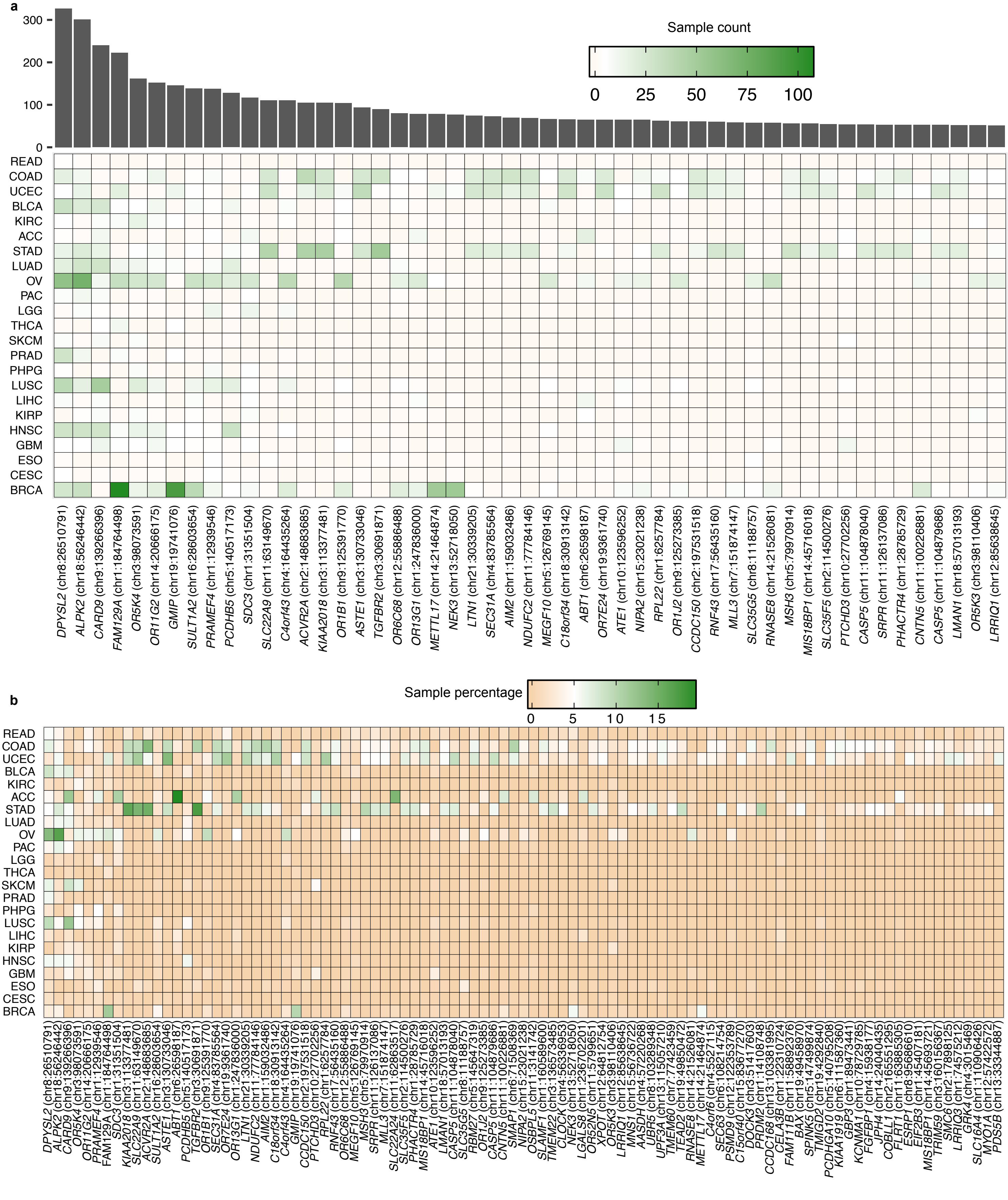


Supplementary Figure 7: Coding MSI loci recurrently subject to frameshift MSI events in the 22 cancer types studied (Table 1). This analysis included MSI-H, MSI-L and MSS tumors. The heatmap shows the fraction of tumors from each cancer type harboring frameshift MSI mutations in MS loci located within the coding sequence of the genes indicated on the x axis. The total count of frameshift MSI events at those MSI loci across all tumor types is depicted in the above barplot.

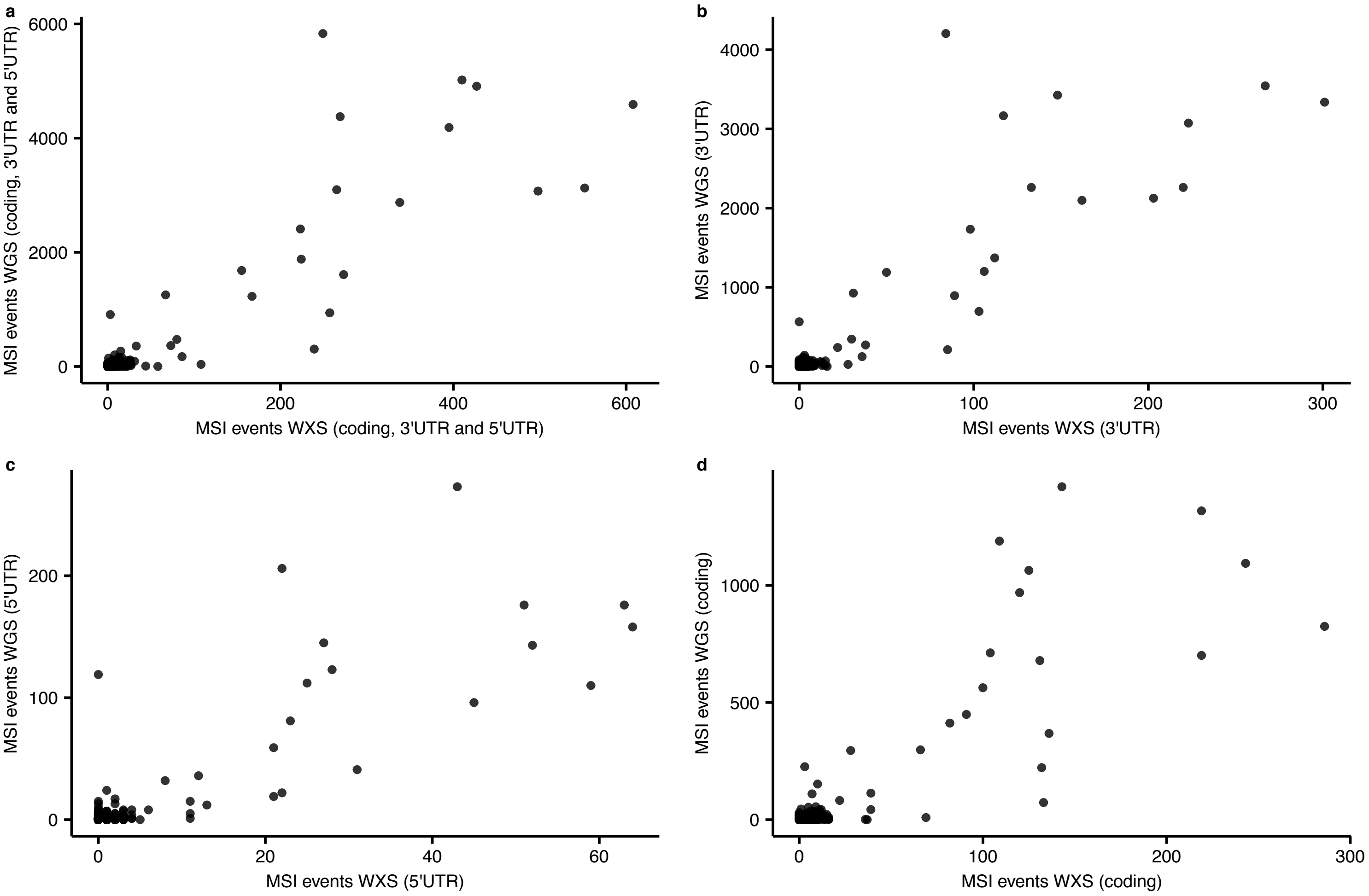


Supplementary Figure 8: Cross-platform comparison of MSI calls in exonic (coding and 3'/5' UTR) (a), 3'UTR (b), 5'UTR (c) and coding (d) MS repeats.

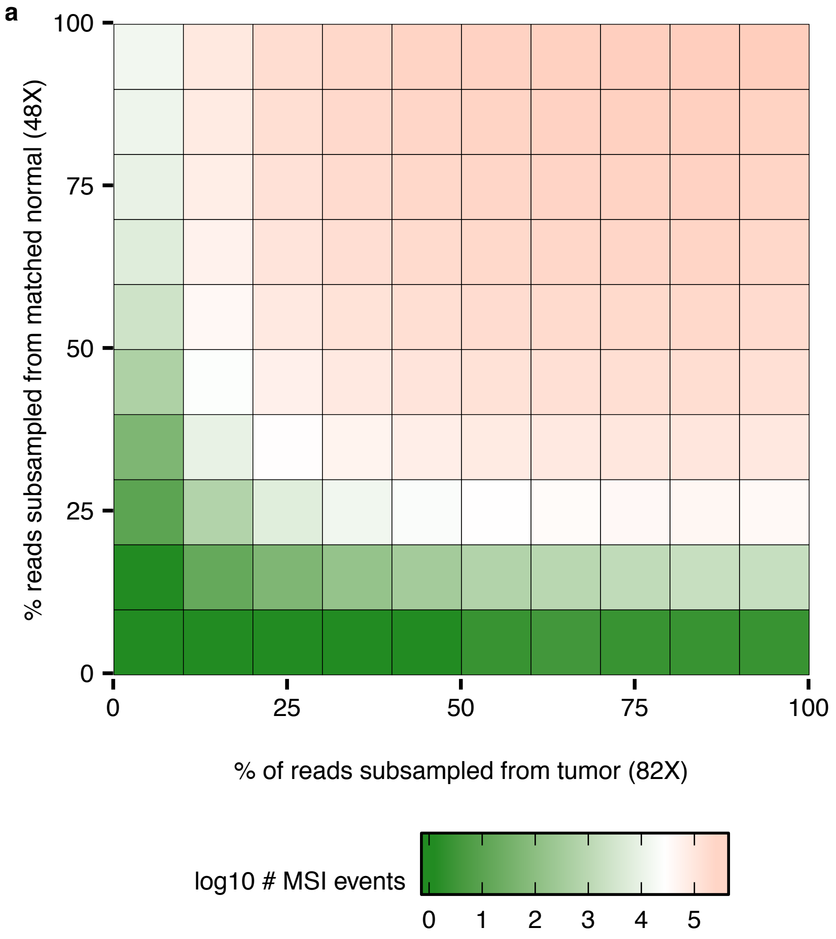


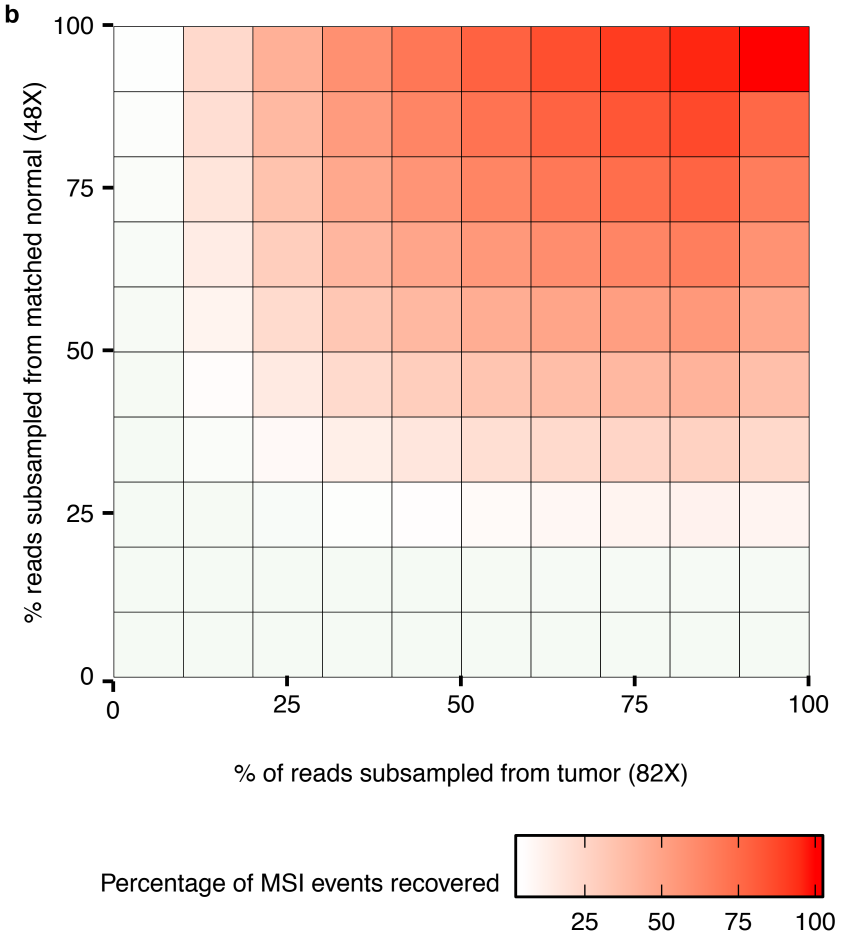


Supplementary Figure 9: Influence of read depth on MSI calling across increasing larger random subsamples of reads from the tumor (82X) and matched normal (42X) bam files corresponding to the patient TCGA-AD-A5EJ. The total number of MSI events and the percentage of MSI called at different subsampling levels are depicted in (a) and (b), respectively.

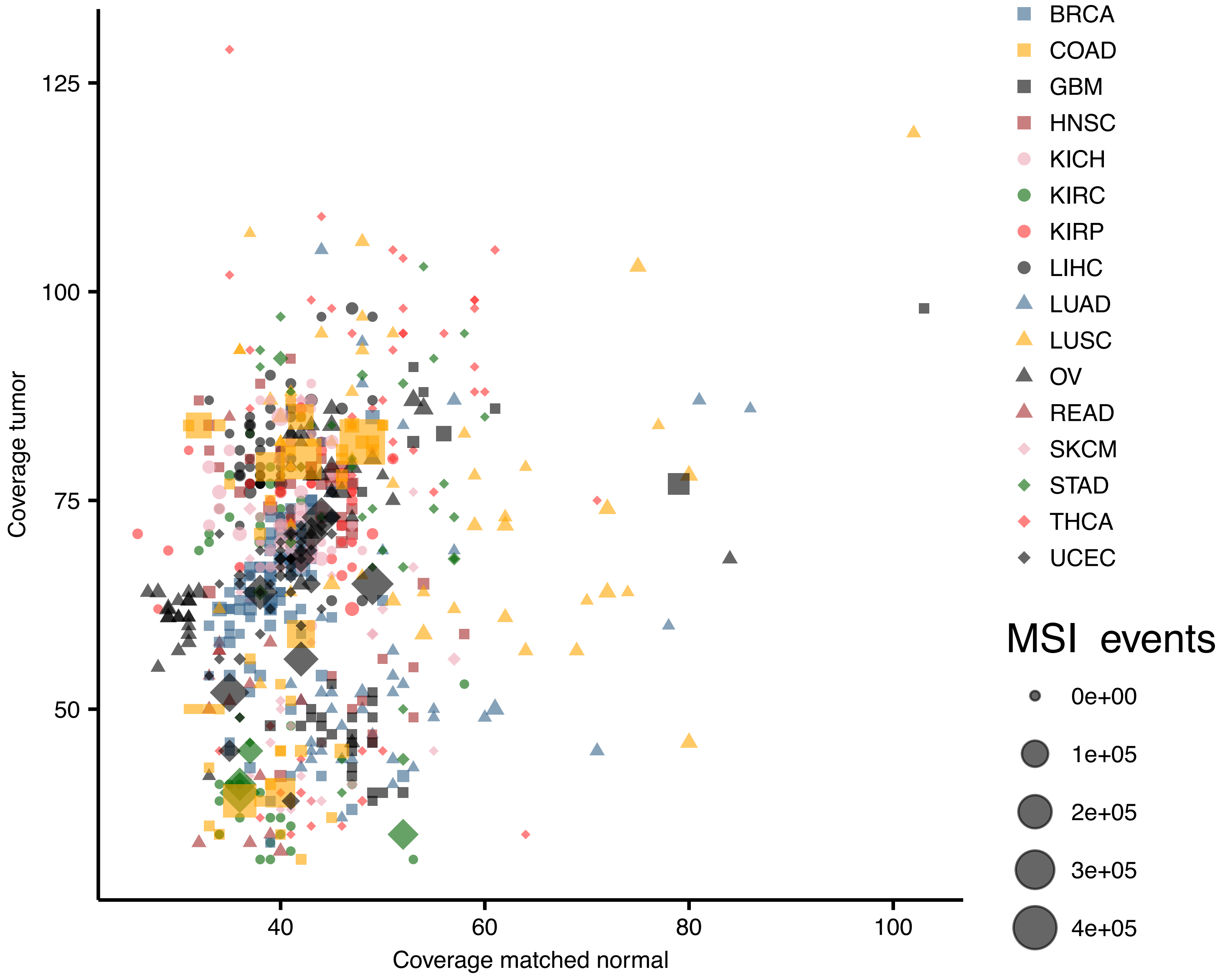


Supplementary Figure 10: Average read depth of the tumor (y-axis) and matched normal (x-axis) samples.

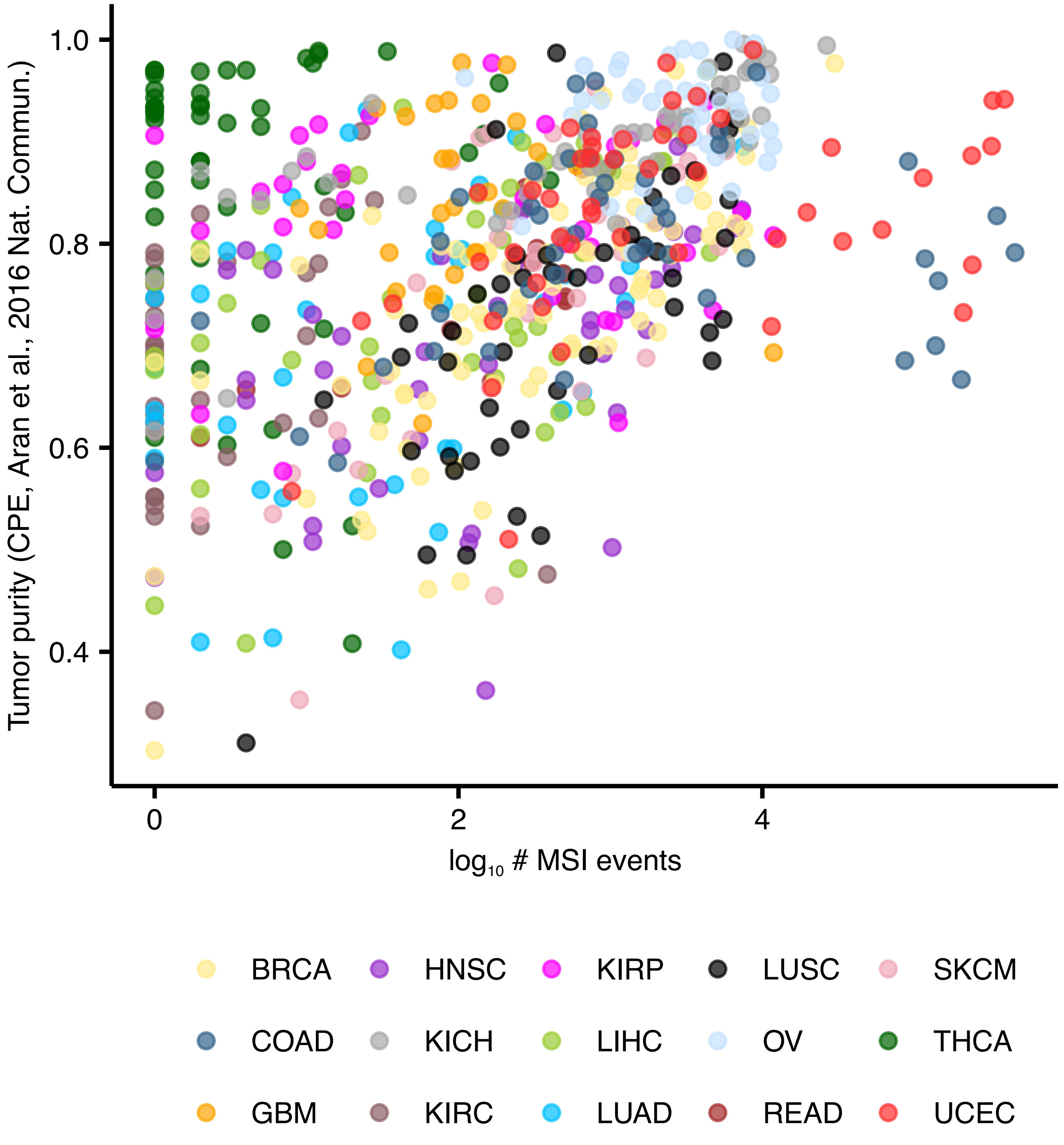


Supplementary Figure 11: Correlation between MSI rates vs tumor purity.

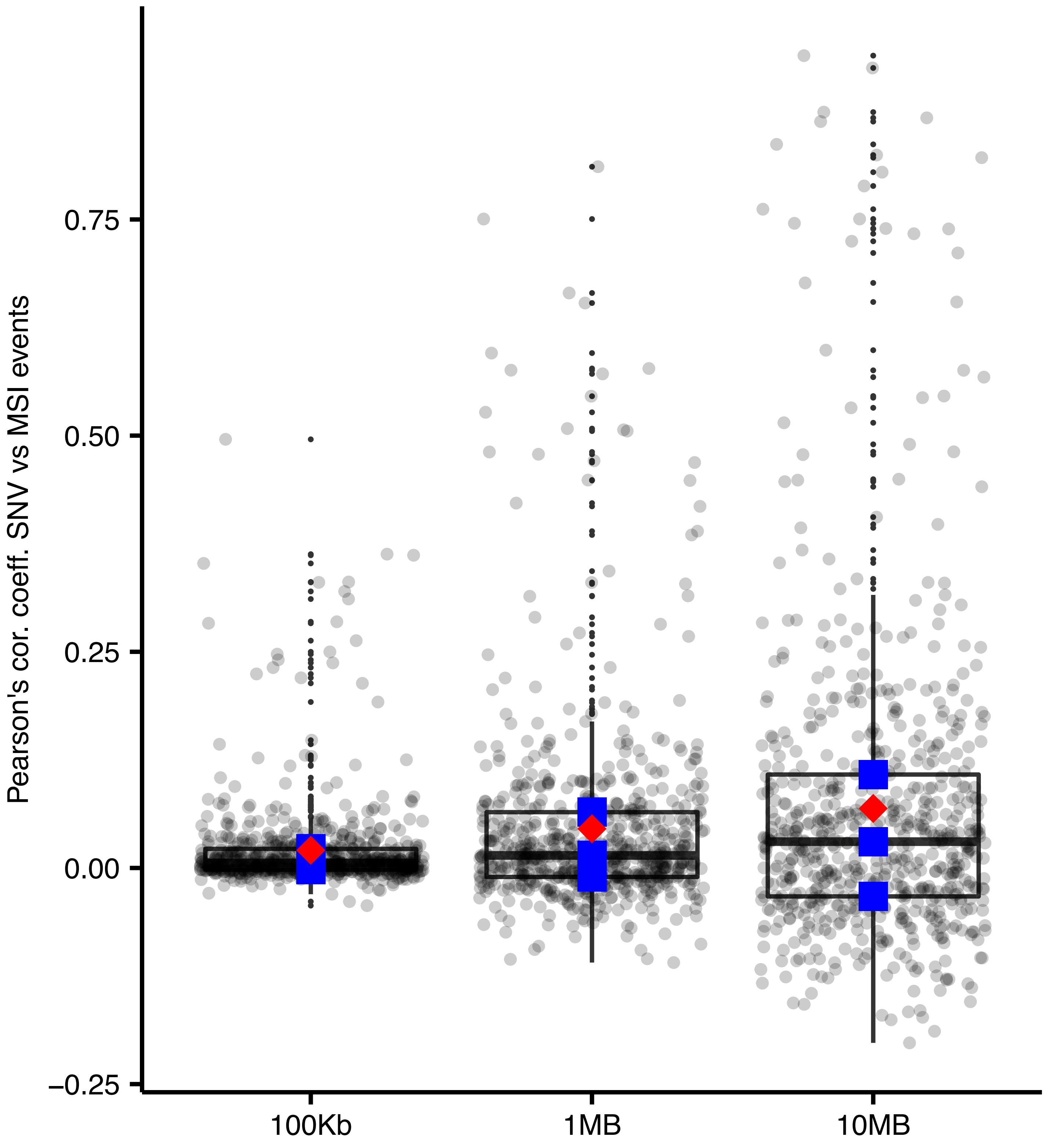


Supplementary Figure 12: Distribution of Pearson correlation coefficients for SNV and MSI rates measured in (a) 1Mb, (b) 10Mb and (c) 100Kb bins. Blue points indicate the median and the interquartile range (25th-75th percentile), whereas red points indicate the mean value.

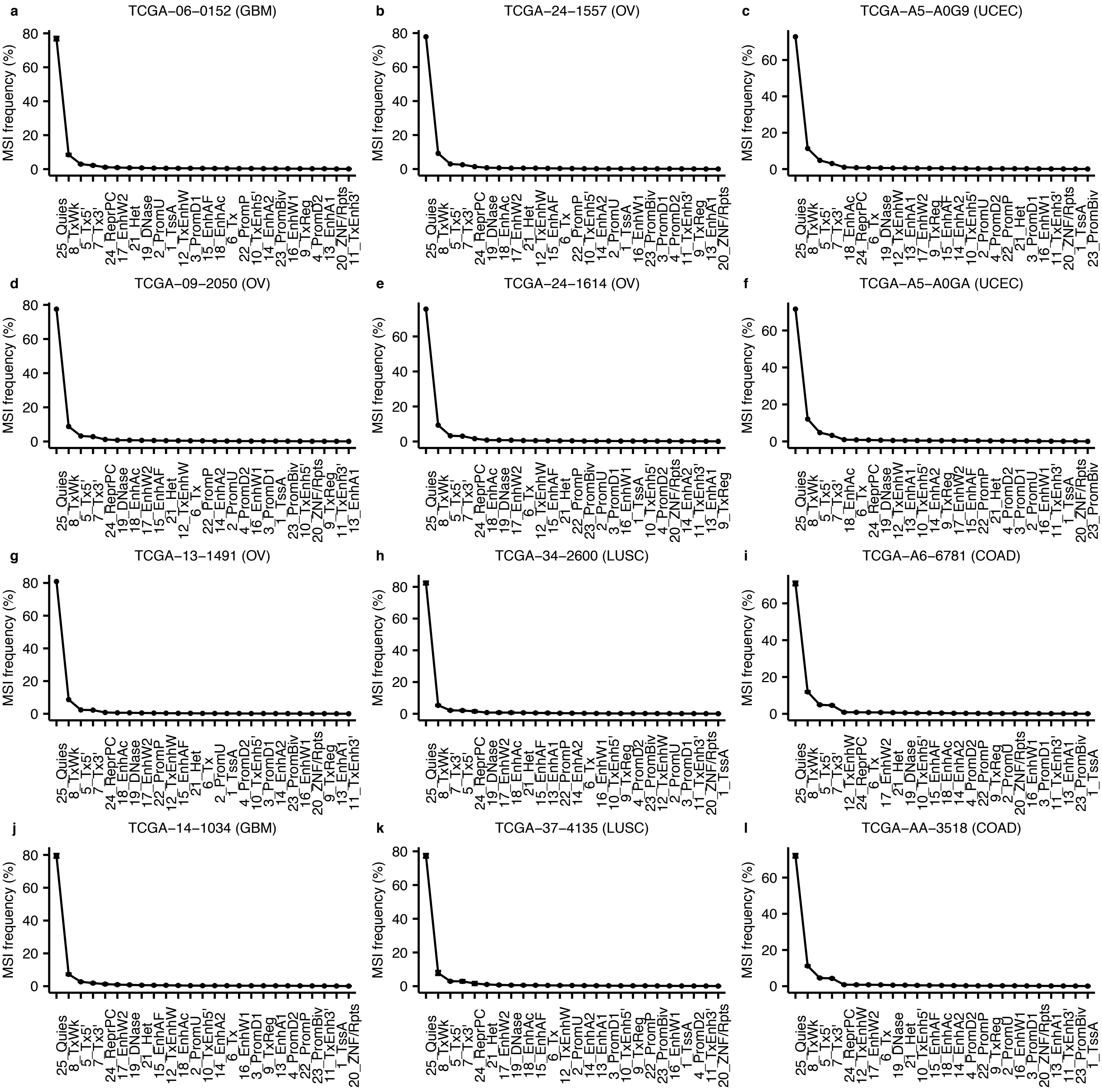


Supplementary Figure 13: Frequency of MSI events in the 25 states of the chromatin state map averaged across 127 epigenomes for samples TCGA-06-0152 (a), TCGA-24-1557 (b), TCGA-A5-A0G9 (c), TCGA-09-2050 (d), TCGA-24-1614 (e), TCGA-A5-A0GA (f), TCGA-13-1491 (g), TCGA-34-2600 (h), TCGA-A6-6781 (i), TCGA-14-1034 (j), TCGA-37-4135 (k) and TCGA-AA-3518 (l).

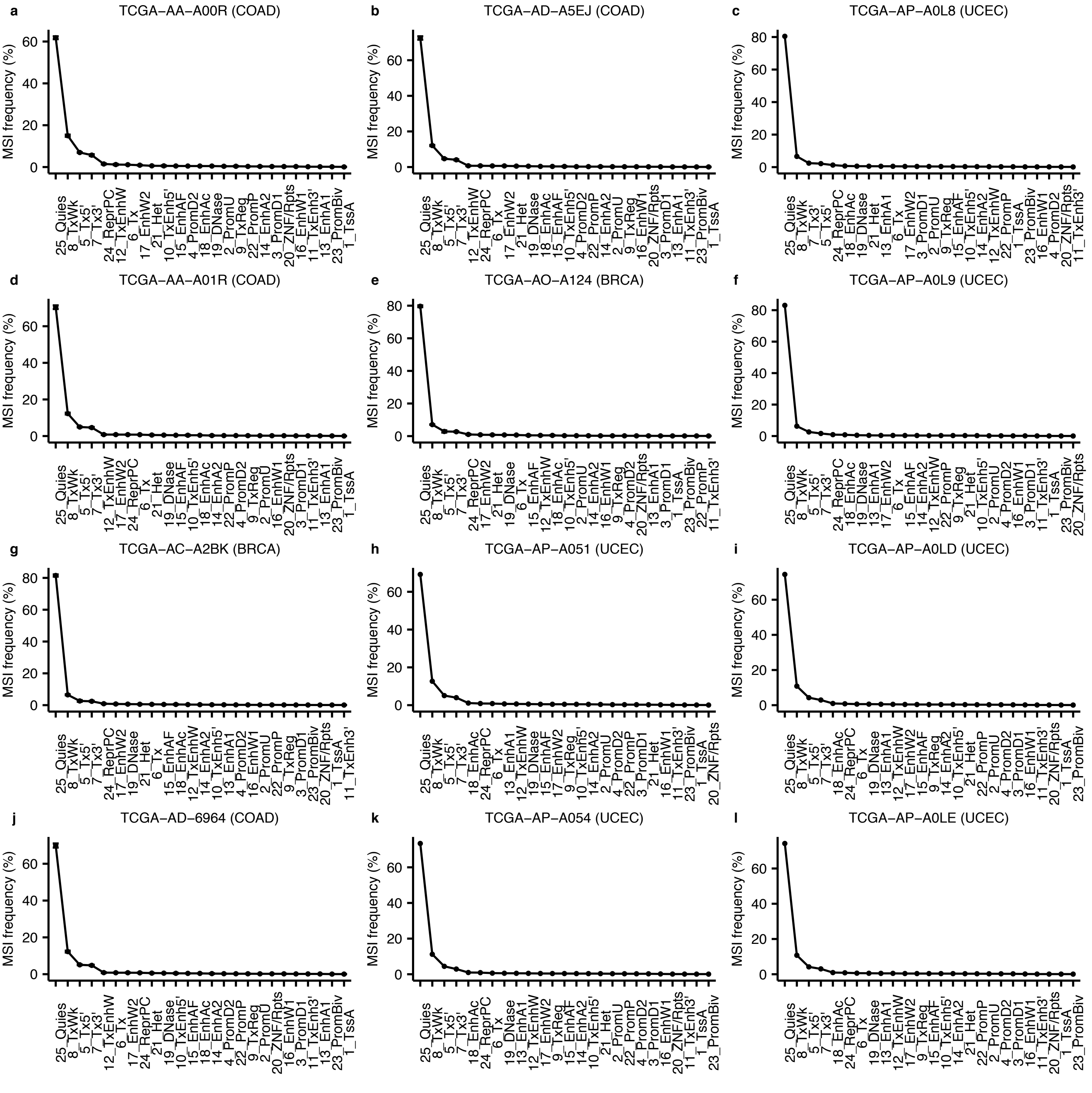


Supplementary Figure 14: Frequency of MSI events in the 25 states of the chromatin state map averaged across 127 epigenomes for samples TCGA-AA-A00R (a), TCGA-AD-A5EJ (b), TCGA-AP-A0L8 (c), TCGA-AA-A01R (d), TCGA-AO-A124 (e), TCGA-AP-A0L9 (f), TCGA-AC-A2BK (g), TCGA-AP-A051 (h), TCGA-AP-A0LD (i), TCGA-AD-6964 (j), TCGA-AP-A054 (k) and TCGA-AP-A0LE (l).

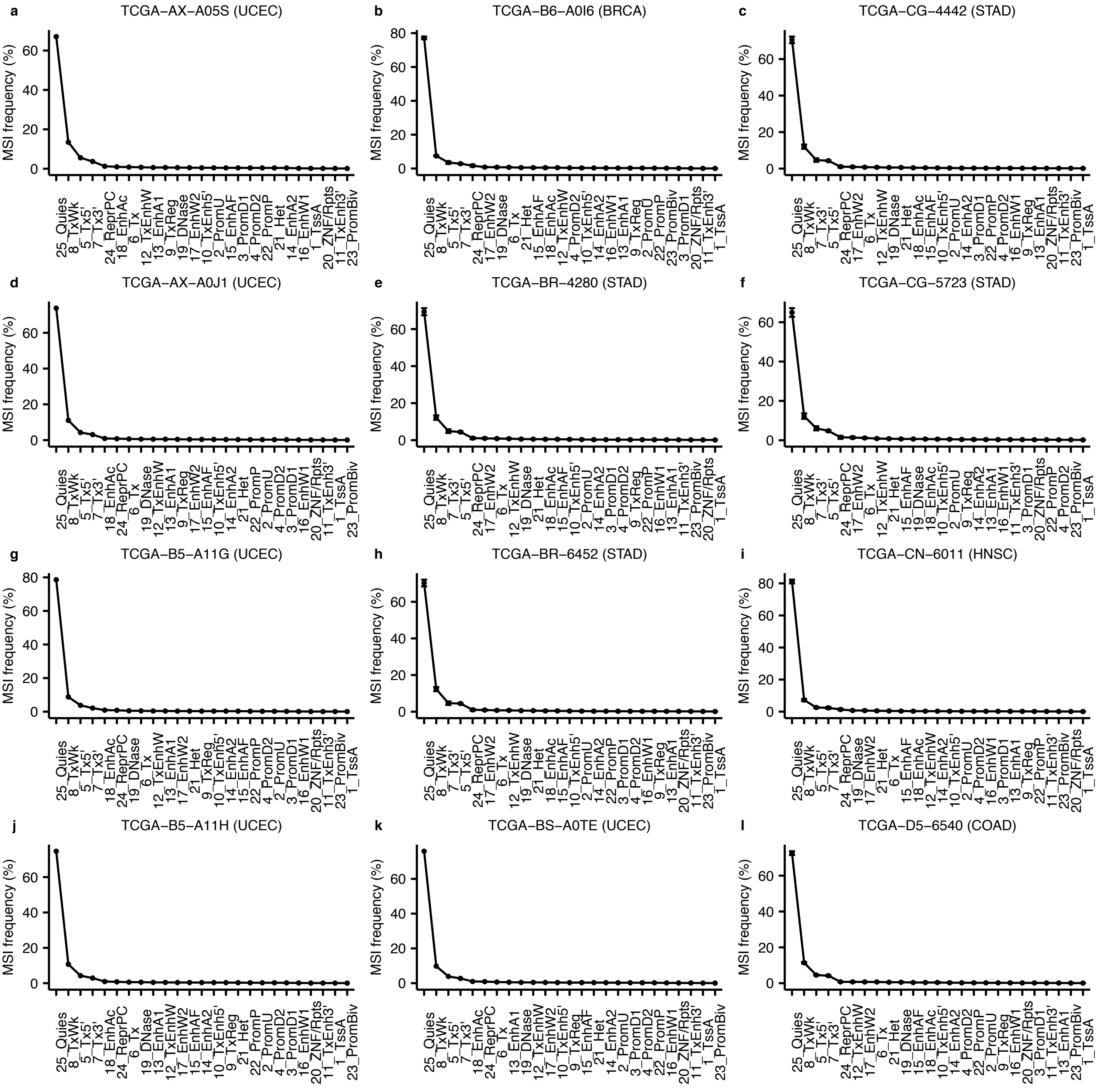


Supplementary Figure 15: Frequency of MSI events in the 25 states of the chromatin state map averaged across 127 epigenomes for samples TCGA-AX-A05S (a), TCGA-B6-A0I6 (b), TCGA-CG-4442 (c), TCGA-AX-A0J1 (d), TCGA-BR-4280 (e), TCGA-CG-5723 (f), TCGA-B5-A11G (g), TCGA-BR-6452 (h), TCGA-CN-6011 (i), TCGA-B5-A11H (j), TCGA-BS-A0TE (k) and TCGA-D5-6540 (l).

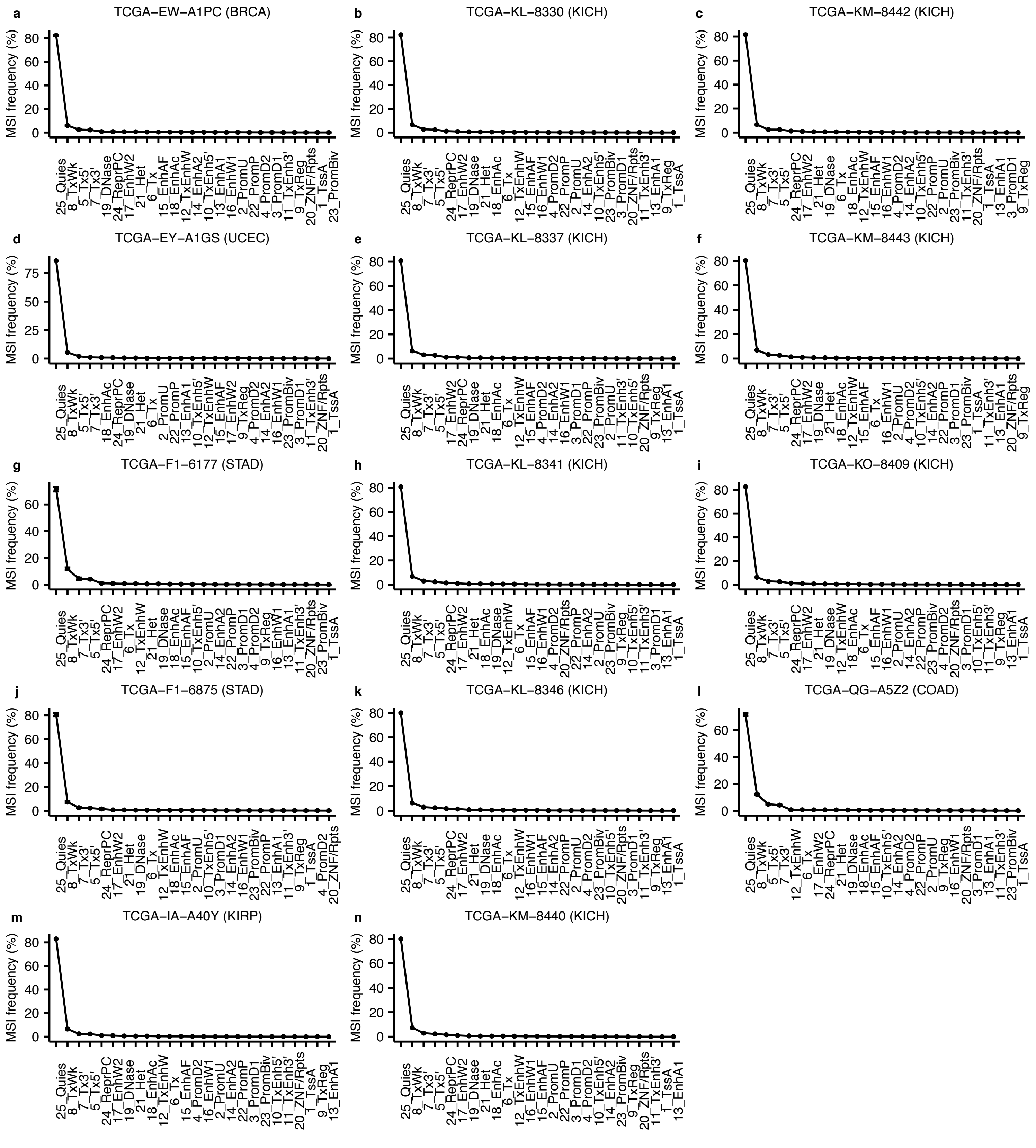

Supplementary Figure 16: Frequency of MSI events in the 25 states of the chromatin state map averaged across 127 epigenomes for samples TCGA-EW-A1PC (a), TCGA-KL-8330 (b), TCGA-KM-8442 (c), TCGA-EY-A1GS (d), TCGA-KL-8337 (e), TCGA-KM-8443 (f), TCGA-F1-6177 (g), TCGA-KL-8341 (h), TCGA-KO-8409 (i), TCGA-F1-6875 (j), TCGA-KL-8346 (k), TCGA-QG-A5Z2 (l), (m) TCGA−IA−A40Y and (n) TCGA−KM−8440.

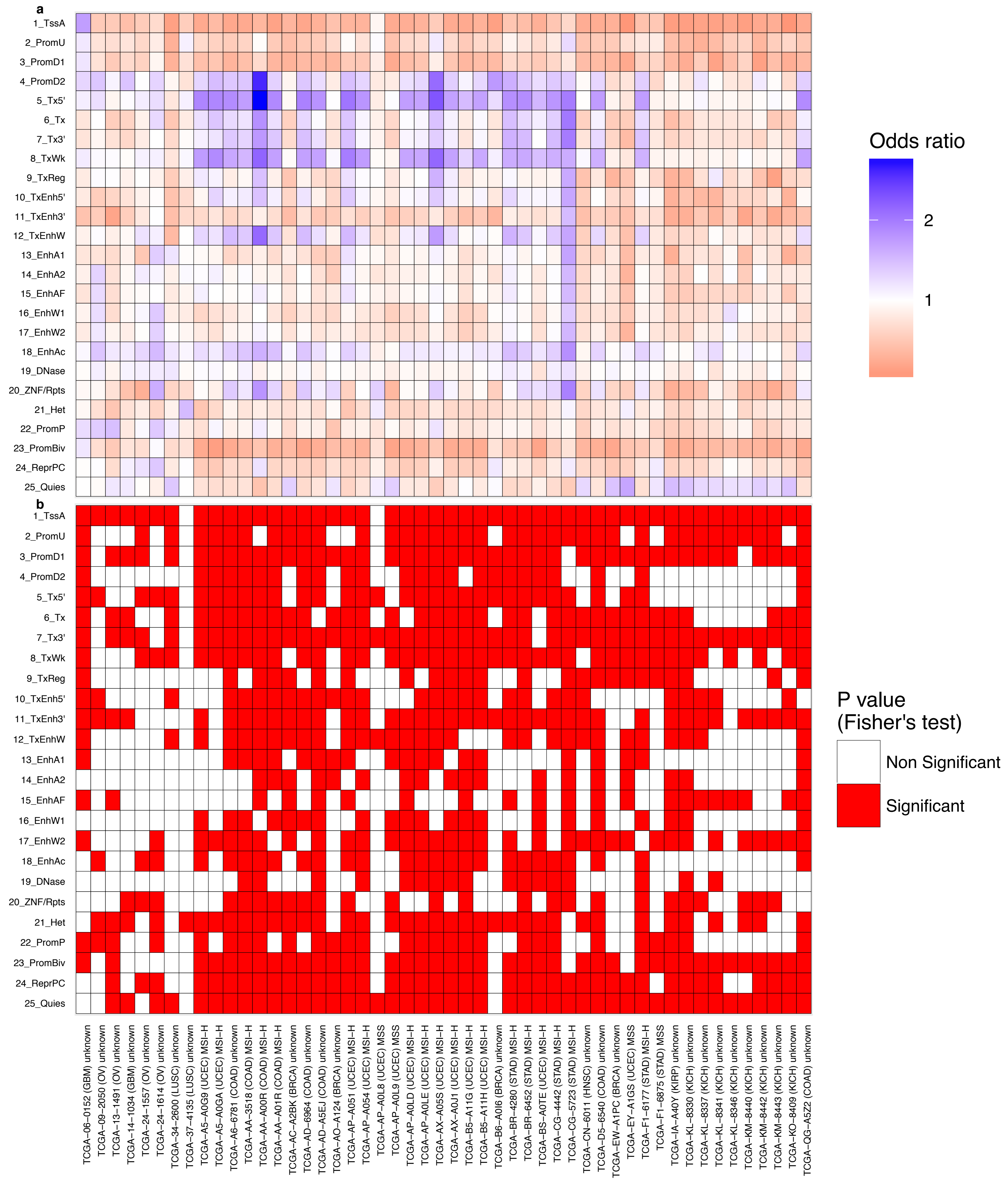


Supplementary Figure 17: Enrichment of MSI events in the 25 states of the chromatin state map. Odds ratio (a) and p values (b) for each chromatin state in the 30 whole-genomes displaying the highest MSI rates. Enrichment of MSI events in each chromatin state was tested using Fisher's exact test (Methods).

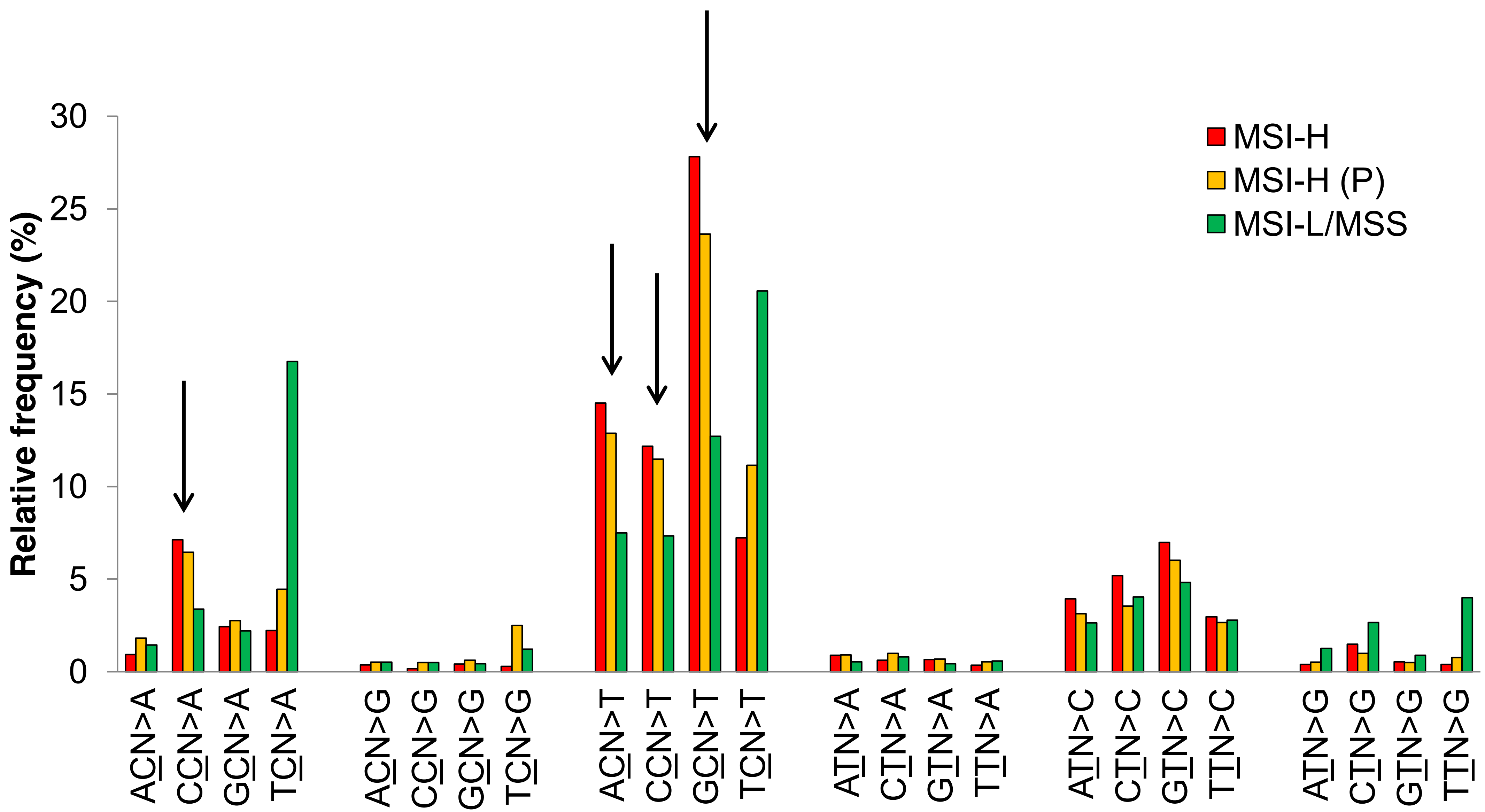


Supplementary Figure 18: Mutation signatures of predicted MSI-H genomes. The relative frequencies of the 96 trinucleotide mutation contexts (strand symmetric) are shown for MSI-H, MSI-H (predicted) and MSI-L/MSS cases (red, orange and green, respectively). Mutations were pooled across the genomes of three MSI-prone tumor types of COAD/READ, STAD and UCEC. Arrows indicate known mutation features of MSI-H genomes, e.g., C>T transitions in (A/C/G)pCpN sequence contexts and C>A transversions at an CpCpN context, which are enriched in both MSI-H and MSI-H (predicted) genomes compared to MSI-L/MSS genomes.
